# Reduced accumulation of defective viral genomes contributes to severe outcome in influenza virus infected patients

**DOI:** 10.1371/journal.ppat.1006650

**Published:** 2017-10-12

**Authors:** Jasmina Vasilijevic, Noelia Zamarreño, Juan Carlos Oliveros, Ariel Rodriguez-Frandsen, Guillermo Gómez, Guadalupe Rodriguez, Mercedes Pérez-Ruiz, Sonia Rey, Isabel Barba, Francisco Pozo, Inmaculada Casas, Amelia Nieto, Ana Falcón

**Affiliations:** 1 Department of Molecular and Cellular Biology, National Center for Biotechnology, Spanish National Research Council (CNB-CSIC), Madrid, Spain; 2 Network CIBER de Enfermedades Respiratorias (CIBERES), Madrid, Spain; 3 Bioinformatics for Genomics and Proteomics, National Center for Biotechnology, Spanish National Research Council (CNB-CSIC), Madrid, Spain; 4 University Hospital San Pedro de Alcantara, Cáceres, Spain; 5 Hospital Virgen de las Nieves, Granada, Spain; 6 Microbiology Department, University Hospital of Vigo, Vigo, Spain; 7 Microbiology Department, Complejo Hospitalario de Ciudad Real, Spain; 8 National Influenza Center, Centro Nacional de Microbiología, Instituto de Salud Carlos III, Madrid, Spain; St. Jude Children's Research Hospital, UNITED STATES

## Abstract

Influenza A virus (IAV) infection can be severe or even lethal in toddlers, the elderly and patients with certain medical conditions. Infection of apparently healthy individuals nonetheless accounts for many severe disease cases and deaths, suggesting that viruses with increased pathogenicity co-circulate with pandemic or epidemic viruses. Looking for potential virulence factors, we have identified a polymerase PA D529N mutation detected in a fatal IAV case, whose introduction into two different recombinant virus backbones, led to reduced defective viral genomes (DVGs) production. This mutation conferred low induction of antiviral response in infected cells and increased pathogenesis in mice. To analyze the association between low DVGs production and pathogenesis in humans, we performed a genomic analysis of viruses isolated from a cohort of previously healthy individuals who suffered highly severe IAV infection requiring admission to Intensive Care Unit and patients with fatal outcome who additionally showed underlying medical conditions. These viruses were compared with those isolated from a cohort of mild IAV patients. Viruses with fewer DVGs accumulation were observed in patients with highly severe/fatal outcome than in those with mild disease, suggesting that low DVGs abundance constitutes a new virulence pathogenic marker in humans.

## Author summary

Influenza A viruses are the causative agents of annual epidemics, sporadic zoonotic outbreaks and occasionally pandemics. Worldwide, acute respiratory infections caused by influenza A viruses continue to be one of the main causes of acute illness and death. The appearance in 2009 of a new H1N1 pandemic influenza strain reinforced the search to identify viral pathogenicity determinants for evaluation of the consequences of virus epidemics and potential pandemics for human health. Here we identify a new general virulence determinant found in a cohort of severe/fatal influenza virus-infected patients, a reduced accumulation of viral defective genomes. These molecules are incomplete viral genome segments that activate the innate immune response. This data will contribute to the prediction of influenza disease severity, to improved guidance of patient treatment and will enable the development of risk-based prevention strategies and policies.

## Introduction

Acute respiratory infections are a main cause of severe illness and death worldwide. Influenza A virus (IAV) causes annual epidemics and occasional pandemics with potentially fatal outcome [1]; the global burden of seasonal influenza is >600 million cases, with 5 million cases of severe illness and up to 500,000 deaths each year. Annual influenza epidemics affect all age groups, although infants, the elderly, and individuals with underlying medical conditions are most severely affected. The existence of co-morbid conditions and the immune status may contribute to the patient outcome. Comorbid conditions for influenza include diabetes, chronic metabolic of lung, renal and cardiac diseases, immunosuppression, pregnancy and obesity [2–4]. Although comorbidities are found in many severe or even fatal cases, a considerable number of apparently healthy individuals nonetheless suffer severe infection, which suggests the coexistence of influenza strains with increased virulence among circulating viruses.

We previously tested this hypothesis by characterizing two IAV strains from the AH1N1 2009 pandemic (AH1N1pdm09), one isolated from a fatal case in a person with no known previously described comorbidities (F-IAV, fatal-case IAV) and the other from a patient with mild symptoms (M-IAV, mild-case IAV) [5]. F-IAV virulence was greater than that of M-IAV in cell culture, and showed higher pathogenicity in the *in vivo* murine model [5].

IAV virulence and pathogenesis are dependent on complex, multigenic mechanisms involving the viral genetic characteristics, the host conditions, the virus-host interactions, and the host response to the infection. Special effort has been previously made to identify virulence determinants of AH1N1pdm09 viruses and AH1N1 seasonal viruses. As a result, some residues distributed all over the genome have been associated to increased virulence of specific viral isolates [6, 7]. These determinants map mainly to the polymerase genes (PB1, PB2, PA), the hemagglutinin (HA), neuraminidase (NA), and non-structural protein 1 (NS1) (reviewed in [8]). Attenuating factors have also been described in cell culture [9, 10].

A proportion of influenza virus particles have defective genome RNAs (DVGs) due to internal deletions of viral segments [11–14]. The DVGs have the 3’ and 5’ ends of the parental RNA segments, and most have a single, large central deletion that generates viral RNAs of 180–1000 nucleotides [15–17]. DVGs have been found for all viral segments, but most derive from PB2, PB1 and PA RNAs [15, 17, 18]. The presence of DVGs potentiates the host response in cultured cells [19, 20] and in animal models and leads to attenuated infection [21], possibly through recognition of double-stranded RNA by receptors that activate antiviral signaling cascades [19] (reviewed in [22]).

Although our understanding of influenza pathogenesis is considerable, a potential general virulence determinant in humans remains to be identified. Here we used next-generation sequencing (NGS) to evaluate the role of defective genomes in the pathogenicity of influenza virus circulating in the human population. We found that the low amount of DVGs accumulated in tissue culture cells correlates with increased pathogenicity in mice both, in natural isolates or recombinant viruses. To corroborate these findings we performed a genomic analysis of viruses isolated from respiratory samples of a select cohort of IAV A(H1N1)pdm09-infected patients who suffered severe or fatal outcome, or from a cohort of infected patients with mild disease. The former viruses showed significantly less accumulation of DVGs than the latter. We suggest that low DVGs abundance has a major role in the severe outcome of IAV-infected patients.

## Results

### Virulence and pathogenicity of F- and M-IAV correlate with DVGs abundance

We previously characterized two virus isolates, the F-IAV derived from a fatal case in a young person with no known previously described comorbidities and the M-IAV, from a young patient with mild symptoms.

#### History of viruses

F- and M-IAV were isolated from upper respiratory tract samples of the infected patients. The viruses were grown in cell culture and in normalized conditions to eliminate possible differences due to the clinical status, time of sampling and genetic background of the infected patients. For that, viral stocks of F- and M-IAV were generated by infection of cell cultures at a low multiplicity of infection (0.0001 moi) to limit the production of DVGs [23] and viruses obtained from this passage were used for the following assays.

#### DVGs accumulation of F- and M-IAV in cell culture

**Fig 1:**
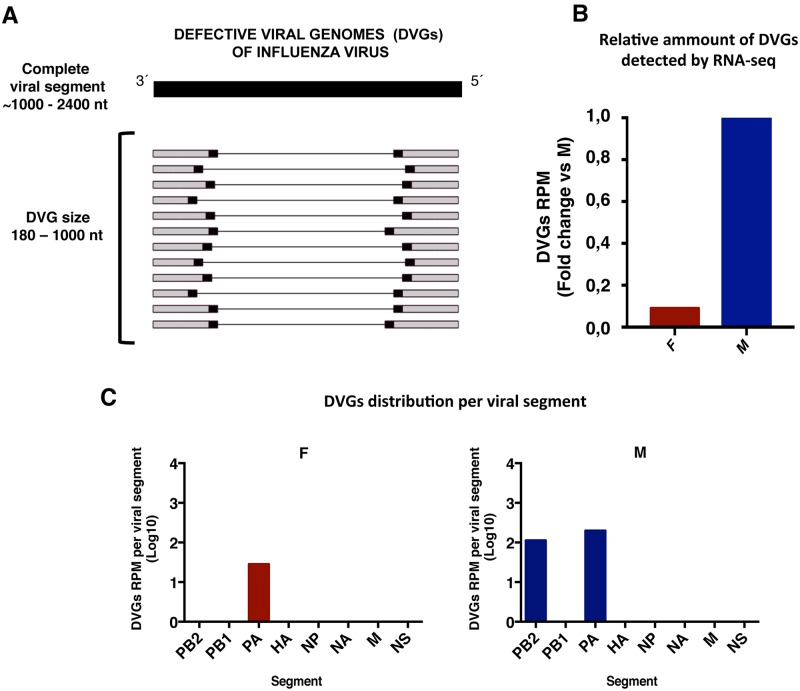
Different amounts of defective genomes are detected in viruses from a fatal or a mild case. (A) Scheme of influenza virus defective genomes (DVGs). Black squares denote the jumping reads. (B) DVGs proportion, calculated as jumping reads/ reads per million (RPM) that align the viral genome, determined in purified virions from a fatal (F) or a mild case (M) of IAV 2009 pandemic infection. DVGs proportion of M-IAV is taken as 1 and fold change for F-IAV is shown. (C). Log scale representation of DVGs distribution per segment calculated as jumping RPM that align each viral segment, analyzed in purified virions from F- and M-IAV. Viral segments, PB1, PB2, PA, HA, NP, NA, M, NS.

**Fig 2:**
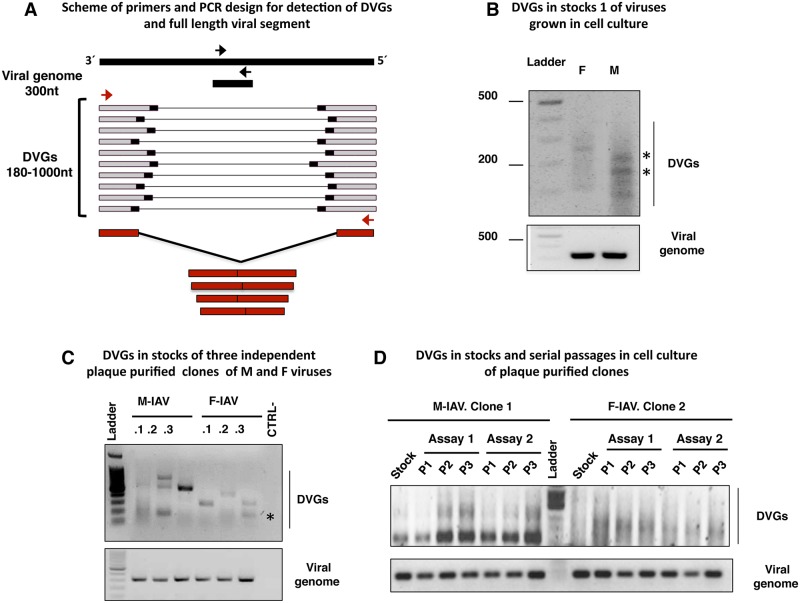
Defective genomes are produced during influenza virus infection and are incorporated into virions. (A) Primers used in PCR to detect the viral genome (black) or DVGs (red). (B) Detection of DVGs (top) or viral genome (bottom) of the PA segment in virions of supernatants from F- or M-infected cell cultures. Asterisks denote bands corresponding to cloned and sequenced DVGs. (C) Detection of DVGs (top) or viral genome (bottom) of the PA segment in virions of supernatants from three independent plaque purified clones (.1, .2, .3) of F- or M-IAV. Asterisk denotes a band corresponding to cloned and sequenced DVG. (D) Accumulation of DVGs after serial blind passages of clone M-IAV.1 and F-IAV.2. Serial passages of each clone were repeated twice (P1-3.1 and P1-3.2). B-D, DNA ladder size indicated in nucleotides.

As described, F-IAV grows faster in cell culture and is more pathogenic in mice than M-IAV [5]. Viral genome RNA obtained from purified virions were deep-sequenced and consensus sequence comparison showed several amino acid changes that might be responsible for their distinct pathogenicity [5]. Detailed analysis of the RNA deep-sequences showed that in addition to the viral full-length RNAs, defective genomic RNAs (DVGs) were observed in these samples (S1 Table). Large internal deletions that generate small DVGs activate the innate immune response [20], and influenza viral RNAs preferably ≤1000 nucleotides (nt) bind the viral sensor RIG-I [19] (reviewed in [22, 24]); thus, only DVGs up to 1000 nt were analyzed in this study (Fig 1A). The proportion between sequences reads corresponding to DVGs deletion sites (junction reads) and total virus reads was calculated for each virus (DVGs proportion); we observed a 10-fold lower DVGs proportion in the F-IAV than the M-IAV (Fig 1B and S6 Fig). DVGs found in F- and M-IAV derived from the RNA segments encoding the viral polymerase subunits as previously described [17] (Fig 1C and S1 Table). To estimate the ability of these viruses to generate DVGs, we analyze the amount of DVGs versus complete viral RNA of the same genome segment by RT-PCR both, in the initial stocks and during the viral replication cycle, performing serial blind passages of F- and M- IAV stocks at 3 pfu/cell. This condition of high multiplicity of infection, would allow an exacerbated generation of DVGs [23]. The results (Fig 2 and S1 Fig), showed that the initial M-IAV stock contained more DVGs than the F-IAV isolate (Fig 2B) and this difference was observed also in three independent plaque-purified clones of F- and M-IAV (Fig 2C). Both, F- and M-IAV accumulated more DVGs along serial passages. However, M-IAV did so more rapidly than F-IAV (Fig 2D and S1A and S1B Fig). The distinct ability to generate DVGs by these viruses was corroborated using two different viral stocks (S1A and S1B Fig), and one plaque purified clone of F- or M-IAV (Fig 2D). Some DVGs amplified by RT-PCR (indicated with asterisks in Fig 2B and 2C) were cloned and sequenced to corroborate that this short amplification products correspond to viral RNA segments with large internal deletions (S2A and S3A Figs).

#### Antiviral response of F- and M-IAV infected cells

To monitor the activation of the cellular antiviral response induced by these viruses, we evaluated the interferon (IFN) induction cascade upon infection of A549 cells that express green fluorescent protein (GFP) under the control of the IFN promoter (A549/pr(IFN-ß).GFP cells). GFP expression acts as a marker for activation of the IFN induction pathway in these cells [25, 26].

**Fig 3:**
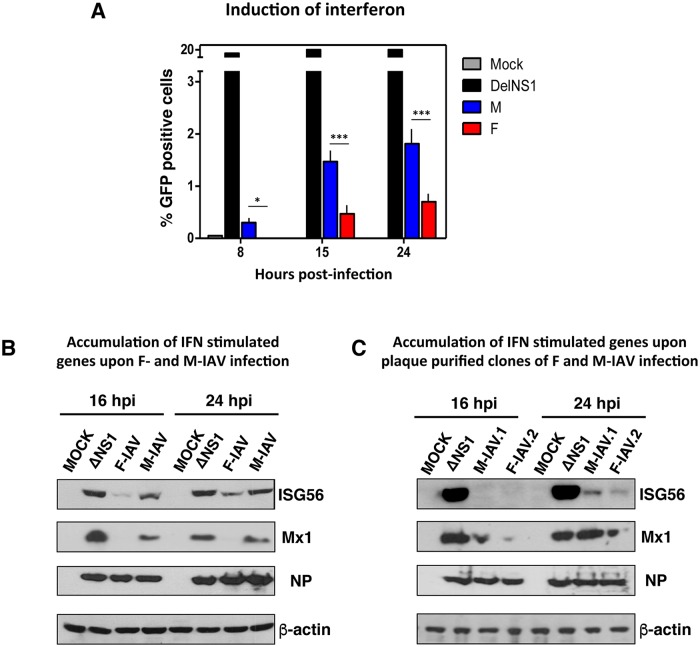
Different induction levels of antiviral response in F- or M-IAV infected cells. (A) Induction of GFP expression after infection of A549/pr(IFN- β) at moi 3.GFP cells were quantified by FACS, using ΔNS1 virus as reference and percentage of GFP positive cells is represented. The results shown are representative of 3 independent experiments, in which 10,000 events were measured for each sample. Significance was determined by two-way ANOVA with Bonferroni post hoc test, *p<0.05; ***p<0.001. (B) Cultured human lung epithelial cells (A549) were infected at moi 3 with F- and M-IAV stocks or (C) with plaque purified clones of F- or M-IAV. At indicated hours post-infection (hpi), samples were used to detect the indicated proteins by Western blot. MOCK, cells treated with PBS as negative control; ΔNS1, cells infected with influenza virus lacking NS1 protein as a positive control of innate immune response activation after influenza virus infection. Virus infection was detected with antibody specific for NP, using β-actin as loading control. The experiments B and C were performed in triplicates and one representative data are shown. Quantification and significance analysis of triplicates is shown in S4 Fig.

These cells were infected with M- or F-IAV at moi 3, or mock infected. As a positive control, cells were infected with Del NS1 virus, an influenza virus that cannot counteract the antiviral response [27, 28]. GFP production was quantified by flow cytometry at 8, 15 or 24 hpi, and the results indicated that DelNS1 virus induces large amounts of GFP and F-IAV was a worse IFN inducer than M-IAV (0.27-fold, p<0.001 at 15hpi and 0.33, p<0.001 at 24hpi) (Fig 3A). To further evaluate the antiviral response mediated by these viruses, A549 cells were infected with DelNS1, M- or F-IAV, or were mock infected, and accumulation of IFN-stimulated genes (ISGs), Mx and ISG56 proteins, was monitored by western blot. F-IAV infected cells accumulated lower levels of Mx and ISG56 proteins than M-IAV (0.29- and 0.48-fold, respectively, p<0.01) (Fig 3B and S4A Fig). The same result was observed for F- and M-IAV clones obtained from purified plaques (0.31- and 0.38-fold, respectively, p<0.001) (Fig 3C and S4B Fig). The difference in the induction of innate antiviral response in infected-human lung epithelial cells coincides with differences in accumulation of DVGs (Figs 1B, 2B–2D and S1B and S1C Fig), which agrees with previous reports [19, 20, 22].

#### DVGs accumulation of F- and M-IVA in lungs of infected mice

Since F-IAV is more virulent than M-IAV in the murine model [5], we tested whether these distinct capacities to generate DVGs were apparent *in vivo*; the accumulation of DVGs and viral genome was analyzed in the lungs of M- and F-IAV-infected mice. In whole lung tissue (S5A Fig) and in virions purified from lung (S5B Fig), DVGs accumulation was greater in M- than in F-IAV-infected mice. There is thus a clear correlation between DVGs accumulation mediated by F- and M-IAV and their pathogenicity in the murine model [5].

### Specific mutations in the polymerase of a fatal-case virus determine DVGs accumulation

Comparison of the F- and M-IAV consensus sequences showed nine amino acid changes in the F isolate [5] taken the A/California/04/2009 strain as reference. Changes in the viral polymerase PA (D529N) and PB2 (A221T) subunits and the surface glycoprotein HA (S127L) found in <1% of viruses circulating during 2009 influenza season were considered specific and potentially responsible for the difference in virulence [5]. Since DVGs are mainly produced by the viral polymerase, PA D529N and PB2 A 221T changes in the polymerase subunits were selected as putative responsible for low DVGs production and increased pathogenicity of F-IAV.

To further characterize the role of mutations in the F-IAV polymerase subunits as virulence determinants, we generated on the A/H1N1/California/04/09 virus backbone (CAL), recombinant influenza viruses bearing the combination of PA D529N and PB2 A221T mutations (PB2/PA *mut*; F-IAV-like polymerase), or viruses bearing single PA D529N (PA *mut*) or PB2 A221T (PB2 *mut*) mutations (Table 1). These viruses were grown in cell culture at a low moi (0.0001) to limit the production of DVGs and viruses obtained from this passage were used for the following assays.

**Table 1:** Amino-acids present in PB2 221 and PA 529 positions in circulating A(H1N1)pdm09 viruses. Fatal-case (F-IAV) and Mild-case (M-IAV) isolates and recombinant viruses generated and analyzed in this study are shown.

**A(H1N1)pdm09 viruses**	**PB2** **221**	**PA** **529**
**Viruses circulating until 2009**	**A**	**D**
**M virus**	**A**	**D**
**F virus**	**T**^**1**^	**N**^**2**^
**2010–2016 circulating viruses**	**A/S/T**	**D**
**Recombinant viruses**	**PB2** **221**	**PA** **529**
**PB2 *mut***	**T**	**D**
**PA *mut***	**A**	**N**
**PB2/PA *mut*** **(F-like polymerase virus)**	**T**	**N**

^1^: Amino-acid found in F virus that was very rare (<1%) in H1N1 viruses circulating up to December 2009, but not in viruses circulating during 2010–2016 influenza seasons. ^2:^ Amino-acid found in F virus which is very rare (<1%) in H1N1 viruses circulating ever until April 2016.

**Fig 4:**
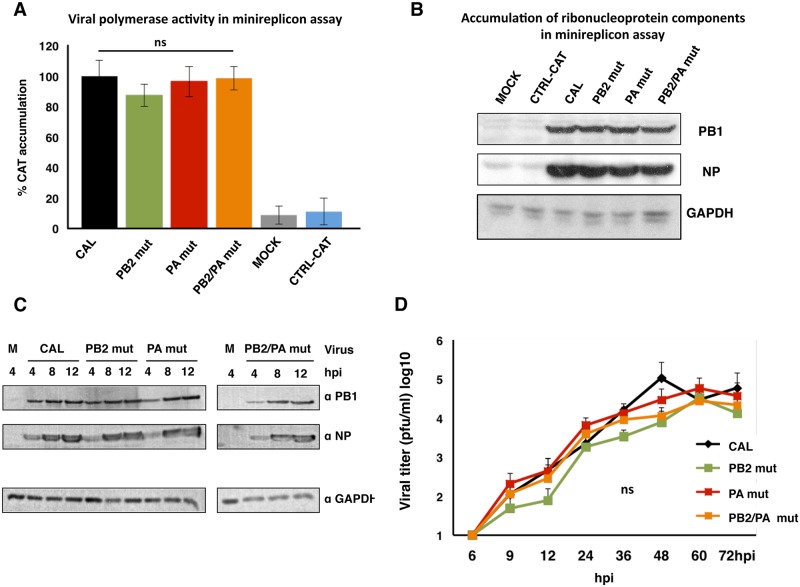
Recombinant viruses have comparable polymerase activity and replicate similarly in cell culture. (A) HEK293T cells were used for *in vivo* CAT RNP reconstitution (see Methods), which indicates viral polymerase activity. At 24 h post-reconstitution, CAT protein in total cell extract was analyzed by ELISA. MOCK, plasmids expressing PB1 or NP were omitted. CTRL-CAT indicates CAT accumulation in cells transfected exclusively with pHHNS-CAT plasmid. Three independent experiments were performed; values shown as means (%) ± SD. Significance was determined by two-way ANOVA with Bonferroni post hoc test (^ns^ not significant). (B) Accumulation of PB1 and NP viral proteins was monitored in cell extracts used for CAT analysis, using GADPH as loading control. (C) Cultured A549 cells were infected at 3 pfu/cell with the recombinant viruses indicated in Table 1. At indicated hpi, samples were used to detect the indicated proteins by Western blot. Three independent experiments were performed and one of them is shown as representative. (D) Cultured A549 cells were infected at 10^−3^ pfu/cell with the recombinant viruses indicated in Table 1. At indicated hpi, supernatants were collected and virus titer determined by plaque assay in MDCK cells. Three independent experiments were performed in triplicate; values shown as means ± SD. Significance was determined by two-way ANOVA with Bonferroni post hoc test (^ns^ not significant).

The activity of the reconstituted polymerases of these three mutant viruses and the wild-type virus was first evaluated in a mini-replicon assay, which showed not significant differences (Fig 4A and 4B). Next, growth kinetics in cell culture showed that all recombinant viruses accumulates similar levels of viral proteins in a single cycle replication assay (Fig 4C) and replicated at a similar rate in a multiple cycle assay (Fig 4D).

#### Deep sequencing analysis of recombinant viruses

**Fig 5:**
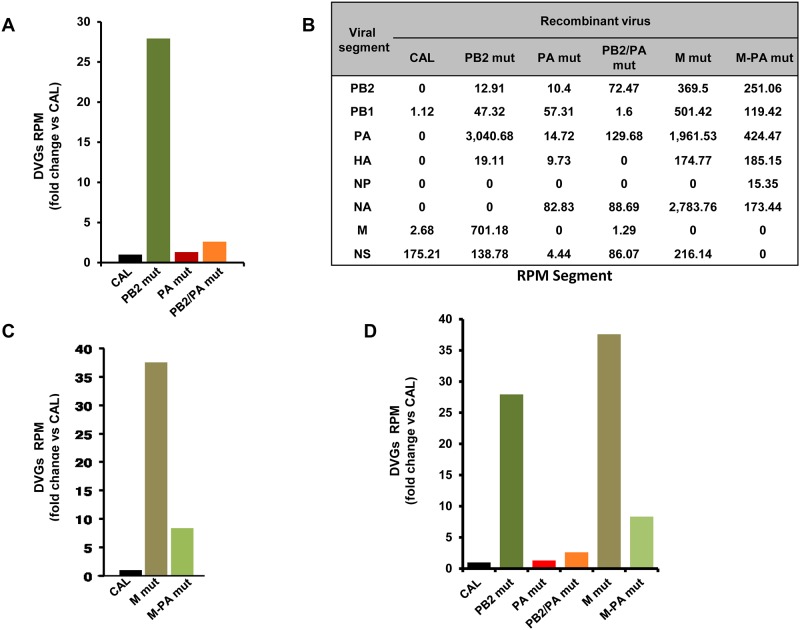
Mutation PA D529N present in a virus from a fatal case reduces DVGs levels in virions of recombinant viruses with two different genetic backgrounds. (A) DVGs proportion calculated as jumping reads/ reads per million (RPM) that align the viral genome, analyzed in purified virions from recombinant viruses generated on the A/H1N1/California/04/09 background. DVGs proportion of the CAL recombinant virus is taken as 1 and fold change is shown for mutant viruses versus CAL. CAL, wild type recombinant virus; PB2 *mut*, recombinant virus bearing PB2 221T mutation; PA *mut*, recombinant virus bearing PA 529N mutation; PB2/PA *mut* recombinant virus bearing PB2 221T and PA 529N (F-like-polymerase) mutations. (B) DVGs distribution per segment calculated as jumping RPM that align each viral segment, analyzed in purified virions from CAL, PB2 *mut*, PA *mut* and PB2/PA *mut*, M *mut* and M-PA *mut* recombinant viruses. (C) DVGs proportion as described in part A, CAL recombinant virus is taken as 1. M *mut* bearing M1 S30N and M2 V86S mutations; M-PA *mut*, recombinant virus bearing M1 S30N, M2 V86S and PA 529N mutations. (D) DVGs proportion, as described in (A) and (C), of all recombinant viruses. CAL is taken as 1 and fold change is shown for the other viruses. Viral segments, PB1, PB2, PA, HA, NP, NA, M, NS.

Deep sequencing of RNA from purified virions of wild-type and mutant recombinant viruses showed that CAL virus accumulates low DVGs levels, which coincides with the reported high virulence of the CAL reference strain [1], and is similar to the F-IAV (S6 Fig). The PB2/PA mut and PA mut viruses showed DVGs accumulation similar to CAL virus (2.6- and 1.3-fold change, respectively; Fig 5A), whereas PB2 mut had a 27-fold higher DVG ratio than CAL virus, which was almost 11-fold higher than PB2/PA mut (with the F-like polymerase) (Fig 5A).

DVGs proportion of CAL virus is taken as 1 and DVGs proportion of the other recombinant viruses are indicated as CAL fold-change. We determined DVGs distribution per viral segment for all DVGs found in each recombinant virus (Fig 5B, left columns), and observed that the DVGs derive from several segments. These data indicated that the PB2 A221T mutation enabled high DVGs accumulation (Fig 5A; compare CAL vs PB2 *mut*), whereas PA D529N mutation restricted their accumulation (Fig 5A; compare PB2 *mut* vs PB2/PA *mut*). The combination of PA and PB2 changes, which mimics F-IAV polymerase, leads to low DVGs accumulation, as also observed for the F-IAV itself.

To confirm that the PA D529N mutation in F-IAV has a major effect on the ability to produce low DVGs numbers, we generated recombinant CAL viruses bearing mutations in genes other than polymerase subunits, such as matrix 1 (M1) and matrix 2 (M2) viral genes (M1 S30N + M2 V86S). These mutations enable influenza virus to accumulate large numbers of DVGs in an H3N2 viral strain [20]. We deep-sequenced RNA from virions purified from recombinant viruses with these M1/M2 mutations, alone (M *mut*) or with these M1/M2 mutations and PA D529N mutation (M-PA *mut*). DVGs number and distribution in these virions are shown in Fig 5C and 5B, right columns. We found a 37-fold DVGs increase in M *mut* compared to wild-type (wt) CAL virus (Fig 5C), in accordance with the ability of the M1/M2 mutations to increase DVGs in an H3N2 influenza virus [20]. As predicted, introduction of the PA D529N mutation on the M *mut* background decreased the DVGs proportion (4-fold; Fig 5C, compare M-PA *mut* vs M *mut*).

In addition, complete viral genome analysis corroborated that each recombinant virus bore the specific mutation or combination of mutations, and no compensatory mutations were detected. Therefore, the differences in the accumulation of DVGs are mediated exclusively by the genetically introduced mutations.

Thereby, PA D529N present in a fatal-case virus is able to decrease the amount of DVGs in two different genetic backgrounds (Fig 5D), reinforcing the notion that this mutation itself confers this ability.

#### DVGs accumulation of recombinant viruses in cell culture

**Fig 6:**
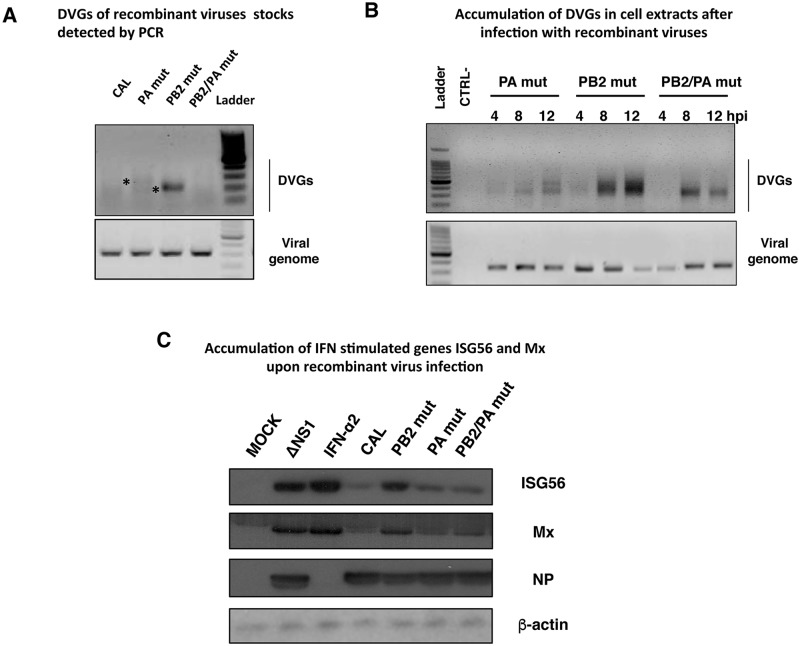
Different amount of defective genomes and activation of antiviral response is produced during recombinant influenza viruses infection. (A) Detection of DVGs of PA segment in virions of CAL, PA *mut*, PB2 *mut* and PB2/PA *mut* recombinant viruses. Asterisks denote bands corresponding to cloned and sequenced DVGs. (B) Cultured human lung epithelial cells (A549) were infected with PB2 *mut*, PA *mut* or PB2/PA *mut* recombinant virus stocks at moi 1. Intracellular accumulation of DVGs was determined at indicated hours post-infection (hpi). DNA ladder size indicated in nucleotides. (C) Cultured human lung epithelial cells (A549) were infected with CAL, PB2 *mut*, PA *mut* or PB2/PA *mut* recombinant virus stocks at moi 1. At 16 hours post-infection (hpi), samples were used to detect the indicated proteins by Western blot. MOCK, cells treated with PBS as negative control; ΔNS1, cells infected with influenza virus lacking NS1 protein as a positive control of innate immune response activation after influenza virus infection. Virus infection was detected with antibody specific for NP, using β-actin as loading control. The experiments B and C were performed in triplicates and one representative data is shown. Quantification and significance analysis of triplicates are shown in S7 Fig.

Similarly to F- and M-IAV, we evaluated DVGs accumulation of viral stocks by analyzing the amount of DVGs versus complete viral RNA of the same genome segment by RT-PCR as described previously (Fig 2A and S2A Fig). This analysis showed that the PB2 mut virus stock had more DVGs than CAL wt virus or virus carrying PA 529N mutation (PA *mut*) and, that PA mutation is able to reduce this large accumulation (compare PB2 *mut* vs PB2/PA *mut* Fig 6A). Some of the RT-PCR amplified DVGs products were cloned (indicated with asterisks in Fig 6A) and sequenced to corroborate that they correspond to viral RNA segments with large internal deletions (S2B and S3B Figs). In addition, cultures of human lung epithelial cells were infected at 3 plaque-forming units (pfu)/cell and accumulation of DVGs over time was evaluated for each mutant virus in these conditions in which the production of DVGs is exacerbated [23]. Total RNA extracts were obtained at 4, 8 or 12 hours post-infection (hpi). The estimation of DVGs amount versus complete viral RNA of the same genome segment was analyzed by RT-PCR as described previously (Fig 2A and S2A Fig). This analysis showed that all viruses generate and accumulate DVGs in the infected cells and PB2 *mut* virus produces DVGs at higher rate than PA *mut* virus (5.4-fold, p<0.05 at 4hpi and 9-fold, p<0.001 at 8hpi) and PB2/PA mut virus (13-fold, p<0.01 at 4hpi and 5.8-fold, p<0.001 at 8hpi) (Fig 6B and S7 Fig).

#### Antiviral response of recombinant viruses

Next, the induction of the antiviral response by the different recombinant viruses was evaluated by monitoring the accumulation of antiviral proteins Mx and ISG56 by western blot at 16 hpi. Treatment with IFN or infection with Del NS1 virus was used as positive control of induction of the antiviral response in these cells. PB2 mut infected cells accumulate higher amount of Mx than the other CAL, PA mut or PB2/PA mut recombinant viruses infected cells (4.4-fold, 2.0-fold and 3.5-fold, p<0.001, respectively) (Fig 6C and S8A Fig). Similar results were obtained for accumulation of ISG56 in PB2 mut infected cells compared to CAL, PA mut or PB2/PA mut infected cells (3.5-fold, 2-fold and 2.3-fold, p<0.001, respectively) (Fig 6C and S8B Fig). There was a clear correlation between the induction of antiviral response (Fig 6C) and the accumulation of DVGs (Fig 6A) as previously described for F- and M-IAV (Figs 1B, 3B and 3C) and for other viruses [19, 20, 22].

### Correlation between DVGs accumulation and *in vivo* pathogenicity

**Fig 7:**
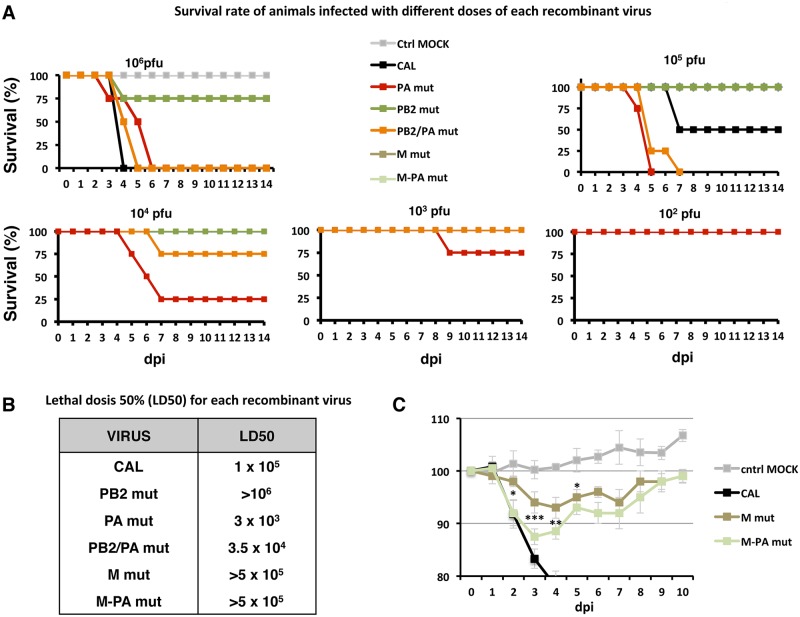
Mutation PA D529N present in a virus from a fatal case increases mortality in vivo. (A) Mice (n = 5) were inoculated intranasally with 10^6^−10^2^ pfu of each recombinant virus or were mock-infected as control. Survival rate for each group of animals was monitored daily for 14 days post-infection (dpi). (B) The dose that caused 50% mortality of mice in each infection (LD50) is shown. (C) Mice (n = 5) were inoculated intranasally with a sublethal dose of each recombinant virus or PBS (MOCK) as control. Body weights were determined daily for 10 days and are shown as a percentage of body weight at inoculation (time 0). Significance was determined by two-way ANOVA with Bonferroni post hoc test (* p <0.05, ** p <0.01, *** p <0.001).

To evaluate the pathogenesis induced by the different recombinant viruses carrying mutations present in F-IAV, we infected mice with various virus doses of CAL, PB2 *mut*, PA *mut* or PB2/PA *mut* viruses or with DMEM as control. Survival (Fig 7A) and body weight (S9 Fig) were monitored daily for two weeks and the lethal dose 50 (LD50) for each virus was determined. CAL, PB2 *mut*, PA *mut* and PB2/PA *mut* viruses showed an LD50 of 1x10^5^, >10^6^, 3 x 10^3^ and 3.5 x 10^4^, respectively (Fig 7B). These data confirmed that CAL virus is pathogenic in mice [1] and indicated that the PA D529N mutation greatly increased pathogenicity, suggesting a decisive effect of this polymerase change on disease outcome. PB2 *mut*, which accumulates high DVG levels, was greatly attenuated compared with the CAL virus. Reconstitution of the F-like polymerase-containing virus (PB2/PA *mut*) notably reduced DVGs accumulation (Fig 5A) and led to higher pathogenicity compared with PB2 *mut* virus (Fig 7A and 7B).

We additionally evaluated the pathogenicity of M mut and M-PA mut viruses in the same way indicated above and the results show that the introduction of the M1+M2 mutations on the CAL wt or PA mut backgrounds led to clear virus attenuation in mice, as the LD50 increased from 1 x 10^5^ or 3 x 10^3^, respectively, to >5 x 10^5^ in both cases (Fig 7B). M-PA mut virus pathogenicity was greater than that of M mut virus, as indicated by body weight loss after sublethal infection (Fig 7C).

**Fig 8:**
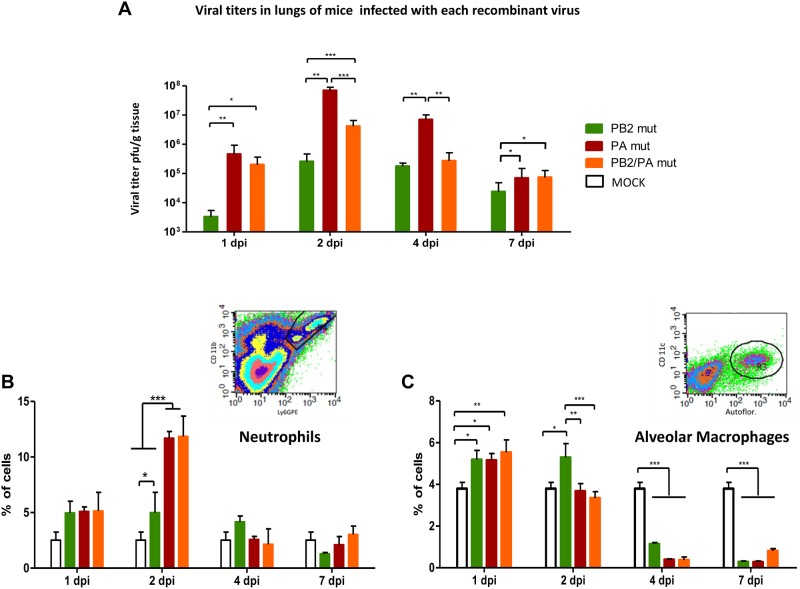
Mutation PA D529N present in a virus from a fatal case increases pathogenicity and induces altered immune response in vivo. Mice (n = 5) were inoculated intranasally with a sublethal dose (10^3^ pfu) of each recombinant virus or PBS (MOCK) as control. At indicated dpi, viral titers were determined in the lungs (pfu/g tissue) (A). Neutrophils (B) and alveolar macrophages (C) were quantified in the lungs (% of cells) at indicated dpi. Significance was determined by Student’s t test (* p <0.05, ** p <0.01, *** p <0.001). Insets show gating information for identification of neutrophil and alveolar macrophage populations.

To further evaluate the pathogenicity differences among viruses bearing mutations present in F-IAV, mice were infected with a sub-lethal dose (10^3^ pfu) of recombinant mutant viruses, or were mock-infected. Samples were recovered at several days post-infection (dpi) and viral titers determined in lung (Fig 8A). PA *mut*-infected mice showed the highest titers at 1, 2, and 4 dpi and the most rapid virus replication kinetics, whereas PB2 *mut*-infected mice showed the lowest titers at all times tested.

#### Immune response of recombinant viruses in infected mice

Virus pathogenicity depends on the nature of the virus, the host conditions and its rapid and adequate response to the infection. We have previously described the higher lung tissue damage induced by F-IAV compared to M-IAV infected mice [5]. Additionally, we have observed that F-IAV seems to induce higher inflammation in the lungs of the infected animals than M-IAV; proliferation of macrophages as a representative lesion of interstitial pneumonia together with vasculitis as accumulation of neutrophils was shown (S10 Fig). To see if the recombinant viruses produced different immune response, we monitored by flow cytometry analysis the presence of neutrophils and alveolar macrophages in PA *mut*, PB2 *mut* or PB2/PA *mut*- infected lungs at several days post-infection of sub-lethal infection (Fig 8B and 8C). Recombinant viruses with PA D529N change (PA *mut* and PB2/PA *mut*) induced higher infiltration of neutrophils and significant depletion of alveolar macrophages than attenuated PB2 *mut* virus at 2dpi in the lungs of infected animals (Fig 8B and 8C). These two alterations in cellular immune response observed for viruses carrying PA D529N change have been described as essential factors for lethal influenza virus infections [29, 30] and agree with the role of PA D529N change in pathogenicity in vivo.

Altogether, these data demonstrate that a mutated PA D529N derived from a fatal-case IAV decreases DVGs generation and support the idea that reduced DG accumulation is a pathogenic determinant for influenza virus in mice. These results corroborate the importance of low DG abundance in fatal influenza outcome.

### Low number of DVGs is associated with severe or fatal outcome in humans

**Fig 9:**
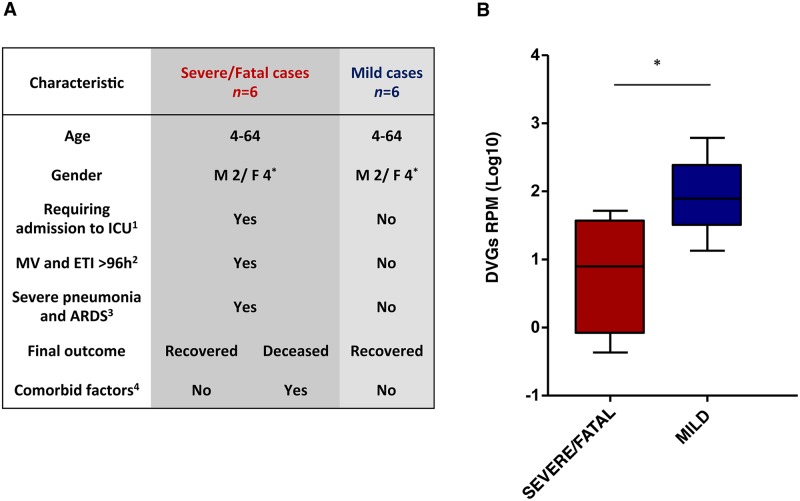
Viruses isolated from severe/fatal-outcome influenza-infected patients have low DVGs numbers. (A) Characteristics of patients included in this study. Male (M), female (F). ^1^: ICU, intensive care unit. ^2^: MV and ETI >96h, requirement of mechanical ventilation and endotracheal intubation for more than 96 hours. ^3^: ARDS, Acute respiratory distress syndrome. ^4^: Comorbid factors include cardiopathy, diabetes, pregnancy, pulmonary disease, immunodeficiency, renal failure, obesity or cardiopulmonary disease. (B) Scatter plot representation of DVGs proportions, calculated as jumping reads/reads per million (RPM) that align the viral genome, found in virions isolated from influenza A virus infected patients. Red and blue boxes indicate the interquartiles with values representing the intermediate 50% of the population. Severe/Fatal cases, n = 6; Mild cases, n = 6. Horizontal bars inside each box indicate the median value. Significance was determined by a two-tailed Mann-Whitney test; p = 0.0152 (* p <0.05).

Next, we wanted to examine whether accumulation of DVGs play a role in the pathogenicity of influenza viruses in humans. Deep-sequencing of RNA from viruses isolated from respiratory samples of a select cohort of A(H1N1)pdm09-like virus-infected patients was performed. This cohort includes patients with highly severe outcome including severe pneumonia and acute respiratory distress syndrome (ARDS) requiring admission to the intensive care unit (ICU) with mechanical ventilation and endotracheal intubation for more than 96 hours (Fig 9A) from 2012–2013 influenza season in Spain. For more precise characterization of the intrinsic pathogenicity of these viruses, only those isolated from patients with no known comorbidities and aged under 65 and over 4 were included (Fig 9A). This cohort (n = 4) is a faithful representation (80–100%) of the total confirmed severe H1N1 influenza cases following these criteria in the 2012–2013 Spanish influenza season (n = 4–5) (S2 Table) [31]. Additionally, two viruses isolated from deceased patients who accomplished these criteria, but otherwise showed underlying medical conditions were evaluated; total severe/fatal cohort n = 6 (Fig 9A). These viruses were compared to those isolated from a cohort (n = 6) of mild IAV patients detected through the regular influenza surveillance system.

#### History of viruses isolated from severe/fatal or mild patients

All these viruses were obtained from exudates of the upper respiratory tract. Similarly to the described above for F- and M-IAV, we isolated the viruses from each clinical sample and the titer of each isolated viruses was determined for this first passage. To obtain the required amounts to purify the corresponding virions, all viruses were grown in cell culture in normalized conditions at a controlled low multiplicity of infection to limit the production of DVGs, as indicated in the material and methods section. This procedure would eliminate RNA amount differences due to the clinical status, time or site of sampling and genetic background of the infected patients and viruses obtained from this passage were used for deep-sequencing (S1 Table).

#### DVGs accumulation of viruses from infected patients

We have obtained the consensus sequence of all mild and severe/fatal viruses and none of the previous DVGs modulating mutations were detected in any of them. Additionally we analyzed in detail the consensus sequence of the three-polymerase subunits for all the evaluated viruses and mutations found in any of the severe/fatal viruses that were not present in mild viruses were selected. The prevalence of these changes in H1N1 viruses circulating in humans was calculated using all sequences available in the NCBI Influenza Resource database up to March 2016. Those mutations present in <1% of the circulating viruses were selected as additional putative severe/fatal exclusive mutations (S3 Table).

**Fig 10:**
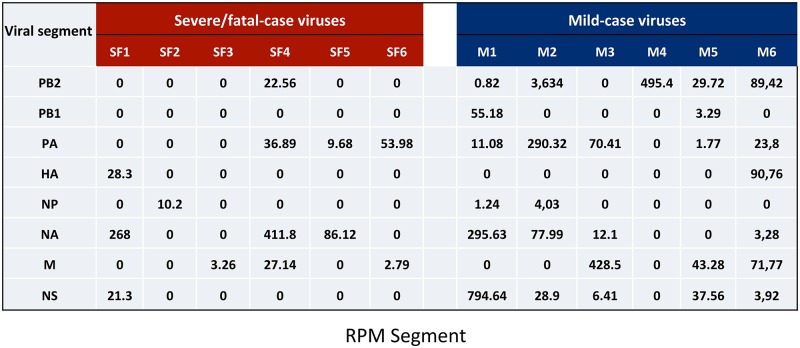
DVGs distribution per viral segment in IAV from highly severe/fatal and mild cases. Absolute numbers of DVGs distribution per segment calculated as jumping RPM that align each viral segment, analyzed in purified virions from A) Severe/fatal-case viruses, SF1-SF6; B) mild-case viruses, M1-M6. Viral segments, PB1, PB2, PA, HA, NP, NA, M, NS. Note that these DGs distributions are relative to all DGs found in each virus, and their amounts are not strictly comparable from one virus to the others.

In addition to the viral full-length RNAs, DVGs were observed in all samples. The proportion between sequence reads corresponding to DVGs deletion sites (junction reads) and total virus reads was calculated for each virus (DVGs proportion); we observed a 10-fold reduction in the DVGs proportion in viruses isolated from severe/fatal cases (n = 6) compared to those from mild patients (n = 6; p = 0.0152) (Fig 9B and S11 Fig). DVGs distribution per viral segment for total DVGs found in each virus is shown for the entire severe/fatal (Fig 10A and S12A Fig) and mild-associated case virus collection (Fig 10B and S12B Fig). Since it has been described that the majority of DVGs are generated in the segments of the viral polymerase [15, 17, 18], we have additionally statistically evaluated the amount of DVGs generated exclusively in those segments (S13 Fig). It can be observed that the difference in these three segments is indeed greater (23-fold; p = 0.0124) than in the whole viral genome. These both data support the notion that viruses with low DVGs abundance have a role in the severe and fatal outcome in IAV infected patients.

## Discussion

Influenza virus pathogenicity has been studied in depth for many years, and several amino acid changes have been identified as virulence determinants [1, 6–8, 32], however, a general pathogenicity determinant has not been characterized. Although DVGs have been described in natural animal infections [33, 34] and in pandemic AH1N1pdm09- and other respiratory virus-infected individuals [35, 36] their role in viral pathogenicity in patients has not been evaluated. The correlation between DVGs accumulation and severe disease observed in the severe/fatal- and mild-case viruses isolated from respiratory samples suggests that DVGs generation is a critical feature of severe influenza virus infection. We tested this hypothesis genetically by analyzing recombinant viruses bearing mutations identified in a fatal-outcome virus (F-IAV, mutations PB2 A221T and PA D529N) [5] or described elsewhere (mutations M1 S30N + M2 V86S) [20]. Whereas the non-pathogenic mutation PB2 A221T accumulates high levels of DVGs in cultured cells and is attenuated in mice (PB2 *mut* versus CAL), mutation PA D529N reduces DVGs accumulation alone or in combination with PB2 A221T change (Fig 5A) or with M1 S30N + M2 V86 S mutations (M-PA *mut* versus M *mut*) (Fig 5C). Moreover, recombinant viruses carrying PA D529N mutation displayed increased viral pathogenicity in the infected mice (PB2/PA *mut* and PA *mut*) (Fig 7).

Selection criteria used for determination of putative virulent markers found in 2009 in F-IAV has been updated, and the prevalence of these changes in H1N1 viruses circulating in humans was calculated using all sequences available in the NCBI Influenza Resource database from December 2009 to March 2016. This analysis showed that PA D529N change continues to be a rare mutation specific of F-IAV, but PB2 221 position admitted several changes including T. This data indicates that at position 221 of PB2 changes have been established in addition to the original A in the further circulating viruses after the influenza 2009 pandemic and this position might not be relevant for the increased pathogenicity of the F-IAV (Table 1). This data correlates with our findings, which indicate that PB2 A221T change is not responsible for the increased pathogenicity of the F-IAV.

Here we adopted a highly restrictive approach to evaluate the potential role of DVGs accumulation as a determinant of severe influenza disease in humans, although a contribution of the immune status of the patient to the infection outcome cannot be excluded. We analyzed influenza viruses from a select cohort of patients, under 65 years and over 4 years of age, who suffered severe or fatal influenza infection. These viruses were compared with those obtained from a cohort of mild infected patients detected through the regular surveillance system. Deep sequencing identified an inverse correlation between DVGs accumulation and virus pathogenicity in these cohorts (Fig 9). Those patients with no known comorbidities infected with low DVGs producer viruses developed a severe outcome, and those who showed comorbid conditions eventually died, indicating that both factors may contribute to the fatal outcome of the infection. The later data is in agreement with our previous study where we found that, besides being infected with the virulent F-IAV, which accumulates a reduced amount of DVGs, the infected patient presented a new confirmed genetic risk factor, a truncated form of *Ccr5* gene [37, 38]. Thus, both the high virulence of the infecting virus, and the genetic risk factor, may have contributed to the fatal outcome of the patient.

None of the severe/fatal cohort viruses bore PA D529N change. This result suggest that low DVGs accumulation in severe/fatal-case viruses might be mediated by various changes other than D529N in PA polymerase subunit, or in distinct polymerase subunits (S3 Table), or in several viral proteins; this coincides with the complex, multigenic nature of the pathogenesis mechanisms [8]. Actually, changes in the polymerase [9] and in NS2 protein [10] modulate DVGs production in cell culture. Mutations in M1 and M2 [20] also modulate DVGs accumulation, probably by altering DVGs encapsidation in progeny virions [39, 40]. Therefore, reduced accumulation of DVGs constitutes a virulent factor itself, regardless the mutations responsible.

Regarding the possible mechanism of PA in low DVGs production, colleagues E. Fodor and G. Brownlee described some years ago that mutation A638R in the PA subunit was involved in the enormous accumulation of DGs in cell culture [9]. It was there described that this high DGs generation was due to an elongation defect by destabilization of RNA-PA subunit interaction, and that this phenomenon could be reverted by another mutation in the same PA polymerase subunit (C453R). The authors proposed a putative domain involved in elongation activity in the PA-C part of this polymerase subunit. We have localized PAD529N mutation in the viral polymerase structure (S14 Fig) [54], and they are spatially in the same PA-C domain nearby these previously described mutations. This data suggests that PA D529N may be involved in the same elongation process, although this activity would need to be further explored. In addition, Influenza A virus polymerase exists in different oligomerization state [55, 56], which allows different assembly of polymerase monomers during replication [57]. A new proposed mechanism suggests that the RNA depending RNA polymerase (vRdRP) dimer bound to viral RNA recruits another free polymerase dimer to form transient tetramer, which initiates replication of viral genome [58]. PA D529N mutation localizes on the interaction surface of this proposed dimerization model of the viral polymerase (S15 Fig). All these data suggest that this mutation (or other mutations on this same area) may alter the possible polymerase dimerization or, it may modify the stability of the RNA- polymerase complex, or additionally it may change the interaction with any cellular required factor, or any combination of them.

The role of DVGs in inducing the innate immune response has been demonstrated in cell culture [19] and animal models [21]. Studies in several animal models have led to proposals for the use of DG molecules or viruses modified to generate them in large numbers as protective elements against influenza virus infection [41]. It would be likely that the reduced activation of the antiviral state of cells infected with low DVGs producer viruses might induce an impaired immune response in infected animals or patients. The initially reduced antiviral state of the infected cells (Figs 3 and 6C) may allow the virus to grow uncontrolled for a short time and then this would induce an exacerbated immune response and inflammation which was described for severe IAV infected patients in the 2009 pandemic [42, 43]. In viral infections, neutrophils and alveolar macrophages play a key role in clearance and control of viral growth in infected lungs, thus their substantial migration to the site of inflammation in infected tissue contributes to overall viral pathogenicity. Depletion of alveolar macrophages leads to an uncontrolled viral proliferation and fatal outcome in infected mice [30, 44] while high influx of neutrophils in lungs and excessive inflammation has been associated with severe illness and high mortality rate in influenza infection [29]. The depletion of alveolar macrophages perfectly correlates with increased viral titers in lung tissue of PA *mut* and PB2/PA *mut* infected animals (Fig 8A and 8C), emphasizing again the crucial role of these cells in viral clearance and control of viral growth. In addition, an increased influx of neutrophils, which is associated with lethal influenza virus infection [29], has been observed in recombinant viruses carrying a mutation (PA *mut* and PB2/PA *mut*) (Fig 8B) present in a fatal-case virus, which produce reduced amount of DVGs (Fig 5A).

Although DVGs are produced in the polymerase segments (PB1, PB2 and PA), as previously described [13, 18], most viruses studied here also generate DVGs in other segments (Figs 5B and 10). The distribution of DVGs of the different cohorts is interestingly observed in some distinct viral segments, which is specially reduced (23-fold) for the polymerase subunits segments in the severe/fatal—associated case viruses compared to viruses isolated from mild cases (Fig 10 and S13 Fig). These findings suggest that the genomic combination of DVGs produced by each virus, and not only absolute numbers, may also contribute to pathogenicity.

In summary, we establish a significant association between low DVGs accumulation and an increase in severe or fatal outcome in human influenza virus infection (Fig 9); we provide genetic support for this association in infected cultured cells and in mice. In addition to the previous reports about the role of DVGs in natural animal infections here we present data indicating that a reduced accumulation of DVGs may be considered a new virulence marker for viral pathogenicity in humans. Evaluation of DVGs phenotype of circulating viruses might predict its potential to induce severe disease. Additional work is needed to define specific DVGs function and the mechanism by which they are produced in humans. These data could contribute substantially to the prediction of influenza disease severity and enable the development of risk-based prevention strategies and policies.

## Materials and methods

### Ethics statement

All procedures that required the use of animals complied with Spanish and European legislation concerning vivisection and the use of genetically modified organisms, and the protocols were approved by the National Center for Biotechnology Ethics Committees on Animal Experimentation and the Consejo Superior de Investigaciones Científicas (CSIC) Bioethics Subcommittee (permit 11014). We followed the guidelines included in the current Spanish legislation on protection for animals used in research and other scientific aims (RD 53/2013) and the current European Union Directive 2014/11/EU on protection for animals used in experimentation and other scientific aims.

The National Influenza Center in Madrid (Instituto de Salud Carlos III) and other regional laboratories from different Spanish regions, constituted the ReLEG network included in the Spanish Influenza Surveillance System (SISS), which monitored the circulation of influenza viruses each influenza season as a part of the countrywide surveillance. The viruses described in this study have been detected within this surveillance activity. An informed consent is not needed for this study since the patients from whom these viruses were isolated were anonymized.

### Biosafety

Cell culture and mouse model experiments performed with recombinant viruses bearing mutations detected in a fatal case of IAV were performed in BSL2+ conditions and in a biological insulator in BSL2+ animal facilities, respectively.

### Biological materials

Cell lines used in this study were canine kidney MDCK (ATCC), human lung epithelium A549 (ATCC) [45] and human embryonic kidney HEK293T (ATCC) cells [46].

### Clinical viral isolates

Viruses used in the present study were selected according to the following criteria for the patients from whom the viruses were isolated. Patients included in the severe/fatal cohort were influenza A(H1N1)pdm09 confirmed cases, aged over 4 and under 65, admitted to intensive care unit (ICU) and with the information related to risk factors reflected in their clinical history. Those patients who developed highly severe disease did not display any comorbidities associated to severe influenza A virus infection, and deceased patients presented some comorbid conditions. Mild patients were influenza A(H1N1)pdm09 confirmed cases, aged over 4 and under 65, who developed mild disease and were monitored by sentinel medical centers included in the Spanish National Influenza Surveillance System. Selection of cases for this mild cohort was randomly made within the patients who meet the described above criteria and whose isolated virus were from the same Saint-Petersburg phylogenetic lineage as those from the severe/fatal cohort, accordingly to their HA gene. Respiratory samples were collected in virus transport medium (MEM, 200 U/ml penicillin, 200 μg/ml streptomycin, 200 U/ml mycostatin and 0.25% bovine serum albumin fraction V) and delivered to the Spanish National Influenza Center.

All influenza A viruses were isolated at the National Influenza Centre (CNM, ISCIII) from respiratory samples sent for virological characterization by the Spanish Influenza Surveillance System (SISS). The National Influenza Center in Madrid and other regional laboratories constitute the ReLEG network of the SISS, which monitors virus circulation each influenza season as a part of the countrywide surveillance. All viruses from either mild or severe/fatal patients were isolated from the upper respiratory tract, pharyngeal or nasopharyngeal exudates. Semi-confluent monolayers of MDCK cells were used for primary viral isolation. The monolayers were inoculated with 200 μl of homogenized samples; when the cytopathic effect was 75–100%, cultures were harvested and the supernatants used for virus stock generation by inoculation of MDCK cells.

### Mutagenesis of pCAGGs-PB2 and PA plasmids derived from CAL/04/09

Specific mutations were engineered in expression pCAGGS plasmids derived from the CAL strain using the QuickChangeTM site-directed mutagenesis kit (Stratagene) as recommended by the manufacturer. These materials were developed using the Licensed technology (Kawaoka-P99264US Recombinant Influenza viruses for vaccines and gene therapy).

### Minireplicon assay

The recombinant minireplicon assay was performed essentially as described [47]. In brief, cultures of HEK293T cells (2.5 × 10^6^ cells) were transfected with a mixture of plasmids expressing the RNP components (pCMVPA, 2.5 ng; pCMVPB1, 12.5 ng; pCMVPB2, 12.5 ng; and pCMVNP, 500 ng) and a genomic plasmid expressing a viral RNA (vRNA)-like chloramphenicol acetyltransferase reporter gene (pHHCAT, 500 ng) using the calcium phosphate technique [48]. At 20 h posttransfection, total cell extracts were prepared and CAT accumulation determined by enzyme-linked immunosorbent assay (ELISA; GE Healthcare), using purified CAT enzyme as a standard.

### Generation of recombinant influenza viruses

Specific mutations were engineered in recombinant virus genomic pHH plasmids derived from the A/H1N1/California/04/2009 strain using the QuickChange site-directed mutagenesis kit (Stratagene) as recommended by the manufacturer. These materials were developed using the Licensed technology (Ref. Kawaoka-P99264US Recombinant Influenza viruses for vaccines and gene therapy). The mutations were rescued into infectious virus by standard techniques [49, 50]. Briefly, to rescue infectious virus from cDNAs, 105 293T HEK cells were cotransfected with a mixture of 12 plasmid DNAs (100 ng each) including (i) 8 genomic plasmids each carrying a viral segment cDNA under the control of the polI promoter and (ii) 4 expression plasmids encoding the three polymerase subunits and the NP. Transfection was carried out at with Lipofectamine Plus (Gibco) under the conditions recommended by the manufacturer. At 16 h post-transfection, transfected cells were plated onto an excess of MDCK cells. When a cytopathic effect was apparent, the supernatant medium was collected and used for plaque assay on MDCK cells to estimate viral titer. The supernatant was used to produce a viral stock at low multiplicity of infection. The identity of rescued mutant viruses was ascertained by sequencing of DNAs derived from the PA and PB2 RNA segments by reverse transcription-PCR (RT-PCR) amplification.

### Generation of viral stocks

Supernatants of harvested cells inoculated with clinical samples or transfected with plasmids for the generation of recombinant viruses were titred by standard plaque assay. These first passages of every virus were used to inoculate fresh MDCK cells at indicated controlled low multiplicity of infection (0.0001 moi). All viral stocks used for further studies had a viral titer about 10^7^ pfu/ml.

### Virion purification and RNA isolation

For virus purification, culture supernatants of 10^−4^ moi-infected MDCK cells were centrifuged (10 min, 3110 *g*, 4°C). Supernatants were sedimented through a sucrose step gradient (TNE buffer; 50% and 33% sucrose in 50 mM Tris-HCl, 100 mM NaCl, 5 mM EDTA, pH 7.5) (1 h, 274000 *g*, 4°C). The 50 to 33% interphase was collected, diluted in TNE buffer, and pelleted through a cushion of 33% sucrose in TNE (2 h, 112000 *g*, 4°C). For purification of viruses isolated from infected mouse lung, the previous protocol was used with modification of the sucrose gradient volume and rotors according to sample volume. For isolation, RNA in purified virions was treated with 0.5% SDS and 200μg/ml proteinase K in TNE (2 h, 37°C), followed by extraction with phenol-chloroform-isoamylalcohol-hydroxyquinolein and ethanol precipitation [51]. DNA was removed by DNAse treatment (Roche) according to manufacturer’s instructions. Quality and quantity of each RNA preparation was monitored using the Agilent 2100 Bioanalyzer (Agilent Technologies) (S2 Table). Appropriate amounts of each sample were analyzed by high-throughput sequencing (see below).

### Western blotting

For the detection of viral and cellular proteins, total cell extracts were collected and Western blot assays were performed as described [49]. Antibodies to GAPDH, β-actin (both from Sigma), ISG56, Mx1 (both from Santa Cruz), NP and PB1 [57] were used.

### Deep sequencing

Sequencing for previously described F- and M-IAV isolated during the 2009–2010 [5] influenza season was performed with the Illumina Genome Analyzer IIx using Illumina v5 sequencing chemistry and 36 bp single reads. Base calling was performed using Illumina pipeline version 1.7.0 (within SCS 2.8). All other viruses were sequenced with TruSeq v3 chemistry and 50 bp single reads on an Illumina HiSeq 2000. Total reads in each sample are indicated in S1 Table.

### Deep sequencing data analysis

#### Short read alignment against the influenza genome

For each sample, FASTQ sequences were aligned against the influenza (A/California/04/09) genome with TopHat2 [52], allowing intron size ranges between 5 and 100000 nucleotides. Samtools [53] was used to extract reads that aligned as two separate fragments (jumping reads) from the BAM files generated by TopHat2. Only reads that were split into two fragments that align at distant segment positions (jumping reads) were selected. Alignments were made using all default parameters with just one exception: we have been more restrictive than the default program and have taken into account just jumping reads supported by at least four nucleotides on one side of the junction. Additionally, only primary alignments were taken into account and secondary alignments proposed by the program were discarded. Reads which align with total viral genome and with each viral segment, and jumping reads in each sample are shown in S1 Table.

#### Viral sequence consensus determination

To determine the consensus sequence of each virus, coverage and nucleotide composition of aligned reads were analyzed. Nucleotide positions with an identity of ≥75% were considered. Samtools mpileup [53] and in-house php scripts were used.

#### Junction quantification, filtering and normalization

Only defective segments generated by two or more jumping reads spanning exactly the same coordinates and that generate a theoretical final RNA segment size of ≤1000 nucleotides were selected. For samples from the year 2009, read orientation could not be determined as the sequencing protocol applied was not strand-specific. In the remaining samples, only reads from genomic RNA strand were counted. For each sample, values for jumping reads per million (RPM) were calculated as the sum of all selected jumping reads divided by the number of total reads that aligned with the influenza genome, then multiplied by 10^6^. For analysis of jumping reads per segment, RPM values were normalized to total reads aligned with each RNA segment of the genome.

#### Nucleotide sequence accession number

The RNA-Seq data obtained in this study have been deposited in the NCBI-SRA database (http://www.ncbi.nlm.nih.gov/sra) under accession no. SRP077920.

### DVGs detection

RT-PCR for the PA or PB2 segments was used to determine the presence of DVGs and their relative amount to the full-length RNA of the same viral segment. To detect full-length segments, internal primers were used to amplify a central fragment, which is not present in DVGs. To detect DVGs, the same RNA sample and external primers of the PA segment were used in a separate reaction. Short amplification times were applied for the detection of both, internal fragment corresponding to full-length segment and DVGs, to allow detection of RNAs up to 1000nt in length. The method is illustrated in Fig 2A. The reverse transcription reaction was performed for 30 min (42°C), followed by PCR (35 rounds at 94°C for 30 s, 53°/58°C for 40 s, and 68°C for 40 sec using the Titan-One RT-PCR kit (Roche)). As a specificity control, the primers and RT-PCR conditions for DVGs amplification were used with a plasmid encoding the full-length PA segment, and no amplification product was obtained (S1A Fig). Additionally, primers and amplification conditions for internal fragment corresponding to full-length segment were used with purified DVGs, and no amplification product was obtained (S1A Fig).

### Cloning and sequencing of DVGs

DVGs from cell cultured purified virions and from infected mice lung tissues were amplified by RT-PCR (Titan-One RT-PCR kit, Roche) as indicated above. Obtained products were amplified with Taq Polymerase (Sigma) for further cloning into pGEM-T vector using pGEM-T Easy kit (Promega). Selected clones were sequenced by Sanger method and obtained sequences were analyzed to confirm that they corresponded to defective genomes, including the 3′and 5′ends and a large internal deletion of the full-length viral segment.

### Virus infections in cell culture

Cultured human lung alveolar epithelial cells (A549) were infected at 10^−3^ pfu/cell (low multiplicity of infection; moi) or 3 pfu/cell (high moi). After 1 h, non-bound virus was rinsed off with acidic PBS (pH 5.3) and at various times (hours post-infection; hpi), cell supernatants were collected and used for virus titration by plaque assay.

### In vivo virus infections

To evaluate pathogenicity of the viruses, 5 female BALB/c AnNHsd mice (6–7 weeks old) were infected intranasally with different doses (10^6^−10^2^) of each of the recombinant influenza viruses described here, or were mock-infected. The animals were monitored daily for clinical signs and body weights for two weeks. For ethical reasons, mice were euthanized when they presented 25% body weight loss.

For the kinetics experiment, 5 female BALB/c mice (6–7 weeks old) were infected intranasally with a sublethal dose (103 pfu/50μl DMEM) of recombinant PA *mut*, PB2 *mut* or PB2/PA *mut* influenza viruses, or were mock-infected (50μl DMEM). Mice were euthanized at 1, 2, 4 and 7 dpi by CO2 inhalation and necropsied.

### Viral titer estimation in extracted organs

Lung samples were homogenized in PBS-0.3%-BSA-penicillin/ streptomycin (100 IU/ml) using an Electronic Douncer (IKA T10 basic, Workcenter). Lung samples were homogenized 1min at max speed at 4°C and debris was pelleted by centrifugation (2000 *g*, 5 min, 4°C). Viral titer was determined by standard plaque assay on MDCK cells.

### Preparation of lung cell suspension

Lungs samples were kept in RMPI medium at 4°C. Tissue samples were grinded into very small pieces prior to digestion with 180 μg/ml *liberase* (*Roche*) and 40 μg/ml *DNase I* (*Roche*) in RMPI medium for 30 minutes at 37°C. Digested fragments were filtrated with *40mm Nylon Cell Strainer* (*BD Falcon*) and resuspended with RMPI- 3%FBS. After centrifugation of samples (1640 rmp, 5 min, 4°C) additional step for erythrocyte lysis were performed. Cell pellet was incubated for 1.5–2 min with 1ml of erythrocyte lysis buffer at RT. Lysis is inhibited by adding 9ml of PBS-5mM EDTA-3%FBS. Samples were then filtrated again with 40mm Nylon Cell Strainer and pelleted by centrifugation (1640rpm, 5 min, 4°C).

### Flow cytometry analysis

Cell suspensions were distributed in 96 wells plate and first incubated with violet LIVE/ DEAD due (*Invitrogen*) for 30 min at 4°C, washed 2 twice with PBS and then incubated for 15 min at 4°C with Fc block CD16 rat antibody. Samples were analysed by staining cell suspension with one or more fluorochrome-labelled antibodies mix in PBS for 30 minutes at 4°C in the dark. Antibodies used were PerCP-Cy5.5-conjugated CD45 (clon 30-F11) (BioLegend), PeCy7-conjugated CD11b (clon M1/70) (BioLegend), APC-conjugated CD11c (clon N418) (eBIOSCIENCE) and PE-conjugated Ly6G (clon 1A8) (BDBIOSCIENCE). Samples were than fixed by incubation with 4% formaldehyde for 20 min, pelleted by centrifugation (700 rmp, 5 min, 4°C) and washed once with PBS. After centrifugation (700 rmp, 5 min, 4°C), cells were resuspended in 0.4ml PBS and kept at 4°C O/N in the dark. Flow cytometric analysis was performed on a cytometer LSR II (BD Biosciences). Data were analyzed using *CellQuestPro* software.

### Histopathology

Animal lungs were fixed in 10% formalin, embedded in paraffin, sliced into 5 mm thick sections, and stained with hematoxylin and eosin (H&E) by conventional methods.

### Localization of mutations in 3D structures

UCSF Chimera 1.10.2 program was used for structural localization of specific mutations in the influenza virus polymerase. Structure of the single influenza A polymerase under accession 4WSB, or structure of the dimerized form of influenza A polymerase complex in Protein Data Bank (PDB) under accession 3J9B have been used as templates.

### Statistics

Student’s *t* test and two-way ANOVA were used as indicated in each experiments and Figures. A non-parametric Mann-Whitney U test was applied to estimate the statistical significance of differences between RPM. GraphPad Prism v. 5.00 (www.graphpad.com) was used for analysis.

## Supporting information

S1 Fig Defective viral genomes are produced during influenza F- and M-IAV infection. (A, B) Accumulation of DVGs after serial passages (P1, P2, P3) of two different M-IAV and F-IAV stocks. DNA ladder size indicated in nucleotides. (C) RT-PCR specificity controls. Primers and amplification conditions for internal fragment corresponding to full-length segment (Int primers) were used with a plasmid encoding the full-length PB2 segment (1 or 1/100 dilution) or with purified DVGs (1 or 1/100 dilution). The primers and RT-PCR conditions for DVGs amplification (Ext primers) were used with a plasmid encoding the full-length PB2 segment (1 or 1/100 dilution) or with purified DVGs (1 or 1/100 dilution).
(TIF)

S2 Fig Alignment of cloned DVGs sequences. PCR amplified DVGs clones of PB2 segment from (A) M-IAV (clones 1 and 2) and F-IAV clinical isolates, or (B) PA mut and PB2 mut recombinant viruses are aligned with the A/Cal/04/09 reference sequence. Dotted lines denote UTR sequences, Red rectangles denote primers for DGs sequencing, Pink rectangles denote PB2 sequences.
(TIF)

S3 Fig Sequence of the DVG clones from PB2 segment. Sequence of the DVG clones corresponding to PB2 segment from (A) M-IAV (clones 1 and 2) and F-IAV clinical isolates or (B) PA mut and PB2 mut recombinant viruses. Underlined sequences represent the primers used for sequencing. Grey boxes come from the 5′end of the PB2 segment fused to the yellow boxes that come from the 3′end.
(TIF)

S4 Fig Quantitative accumulation of antiviral proteins in F-and M-IAV-infected cells. (A) Cultured human lung epithelial cells (A549) were infected with F- and M-IAV stocks or (B) with plaque purified clones of F- or M-IAV at moi 3. At 24 hours post-infection (hpi), samples were used to detect the indicated proteins by Western blot. MOCK cells treated with PBS as negative control and were used as background for quantitative analysis. β- actin antibody was used as loading control. Error bars indicate mean ± SD of three independent experiments (*p<0.05, **p<0.01 by t-Student test).
(TIF)

S5 Fig Defective viral genomes are produced during influenza virus infection and are incorporated into virions in vivo. PCR detection of DVGs (top) or viral genome (bottom) of the PA segment in lung extracts (A) and in virions purified from lungs (B) of mice infected with F- or M-IAV isolates. DNA ladder size indicated in nucleotides.
(TIF)

S6 Fig CAL recombinant virus has as low DVGs numbers as F-IAV. DVGs proportion in CAL recombinant virus and F- and M-IAV clinical isolates. CAL recombinant virus has been used as backbone for mutant viruses in the present study. Scatter plot representation of DVGs proportions, calculated as jumping reads/reads per million (RPM) that align the viral genome. Black indicates CAL wild-type recombinant virus, red indicates F-IAV,-Blue indicates M-IAV.
(TIF)

S7 Fig Quantitative accumulation of DVGs in recombinant virus-infected cells. Cultured human lung epithelial cells (A549) were infected with indicated mutant viruses stocks at moi 1. Intracellular accumulation of DVGs was determined at indicated hours post-infection (hpi) as the ratio between DVGs and full length viral genomes (as shown in Fig 6). Error bars indicate mean ± SD of three independent experiments (*p<0.05, **p<0.01, ***p<0.001 by two-way ANOVA with Bonferroni post hoc test).
(TIF)

S8 Fig Quantitative accumulation of antiviral proteins in recombinant virus-infected cells. Cultured human lung epithelial cells (A549) were infected with CAL or mutant viruses stocks at moi 1. At 16 hours post-infection (hpi), samples were used to detect (A) Mx protein or (B) ISG56 protein by Western blot. MOCK cells treated with PBS as negative control and were used as background for quantitative analysis. β-actin antibody was used as loading control. Error bars indicate mean ± SD of three independent experiments (*p<0.05, **p<0.01, ***p<0.001 by two-way ANOVA with Bonferroni post hoc test).
(TIF)

S9 Fig Mutation PA D529N present in a virus from a fatal case increases pathogenicity in vivo. Mice (n = 5) were inoculated intranasally with different doses (106–102 pfu) of each CAL, PA mut, PB2 miut or PB2/PA mut recombinant virus or were mock-infected as control. Body weight for each group of animals was monitored daily for 14 days post-infection (dpi).
(TIF)

S10 Fig Histological injuries found in lungs of mice inoculated with M- or F-IAV. (A,B) Hematoxylin and eosin staining (H&E) of mice lung inoculated with M-IAV. (A) Congestion and diffuse lymphoid infiltrates in the interstitium. bar = 50 μm, (B) Mild perivasculitis: note mild inflammatory infiltrates of lymphocytes around a small arteriole and diffuse in the interstictial tissue. bar = 50 μm. (C-E); H&E of of mouse lung inoculated with F-IAV. (C) Moderate inflammatory infiltrates of lymphocytes around a dilated bronchiole. bar = 50 μm, (D) Severe hyperplasia of phagocytic cells in the interstitium. bar = 20 μm, (E) Mild perivasculitis: moderate amount of lymphoid cell infiltrates around a pulmonary arteriole. bar = 50 μm. The arrows show some examples of the lesion indicated in every case.
(TIF)

S11 Fig Viruses isolated from severe/fatal outcome influenza-infected patients have low DVGs numbers. DVGs proportions, calculated as jumping reads per million (RPM) that align the viral genome, found in virions isolated from influenza A virus infected patients. Severe/fatal-case viruses, SF1-SF6; Mild-case viruses, M1-M6.
(TIF)

S12 Fig DVGs distribution per viral segment in severe/fatal and mild cases of IAV. Log scale representation of DVGs distribution per segment calculated as jumping reads per million (RPM) that align each viral segment, analyzed in purified virions from A) Severe/fatal case viruses, SF1-SF6; B) mild-case viruses, M1-M6. Viral segments, PB1, PB2, PA, HA, NP, NA, M, NS. Note that these DVGs distributions are relative to all DVGs found in each virus, and their amounts are not strictly comparable from one virus to the others.
(TIF)

S13 Fig Viruses isolated from severe/fatal-outcome influenza-infected patients have low DVGs numbers in the three polymerase subunits segments. Scatter plot representation of DVGs proportions, calculated as jumping reads/reads per million (RPM) that align the viral genome, found in virions isolated from influenza A virus infected patients. Red and blue boxes indicate the interquartiles with values representing the intermediate 50% of the population. Severe/Fatal cases, n = 6; Mild cases, n = 6. Horizontal bars inside each box indicate the median value. Significance was determined by a two-tailed Mann-Whitney test; p = 0.0124 (* p <0.05).
(TIF)

S14 Fig Structural localization of PA mutations in the viral polymerase complex. Structure of the viral polymerase complex in Protein Data Bank (PDB) under accession 4WSB has been used as template for structural localization of PA 529N (red) mutations with UCSF Chimera 1.10.2. PA 638A and 453R mutations previously described [Fodor et al;. 2003] are shown in yellow. RNA viral promoter is shown in blue.
(TIF)

S15 Fig Structural localization of relevant PA mutations in the multi-polymerase complex. Structure of the dimer polymerase complex in Protein Data Bank (PDB) under accession 3J9B has been used as template for structural localization of PA mutations with UCSF Chimera 1.10.2. PA mutations described in the severe-fatal case viruses in the present study (PA 529 and PA 312) are shown in red. RNA viral promoter is shown in blue.
(TIF)

S1 Table RNA quality and jumping reads details. (XLSX)

S2 Table Epidemiological data for 2012–2013 Spanish influenza season. Epidemiological data, including comorbidities information, reported by the National Epidemiological Influenza Center are shown for the 2012–2013 Spanish influenza seasons.
(TIF)

S3 Table Exclusive mutations found in the viral polymerase of the severe/fatal cohort viruses. (TIF)
