# Supplementary figures and images for: Reduced accumulation of defective viral genomes contributes to severe outcome in influenza virus infected patients
Source: PLoS Pathog. 2017 Oct 12;13(10):e1006650. doi: 10.1371/journal.ppat.1006650 (PMC5638565; doi:10.1371/journal.ppat.1006650)

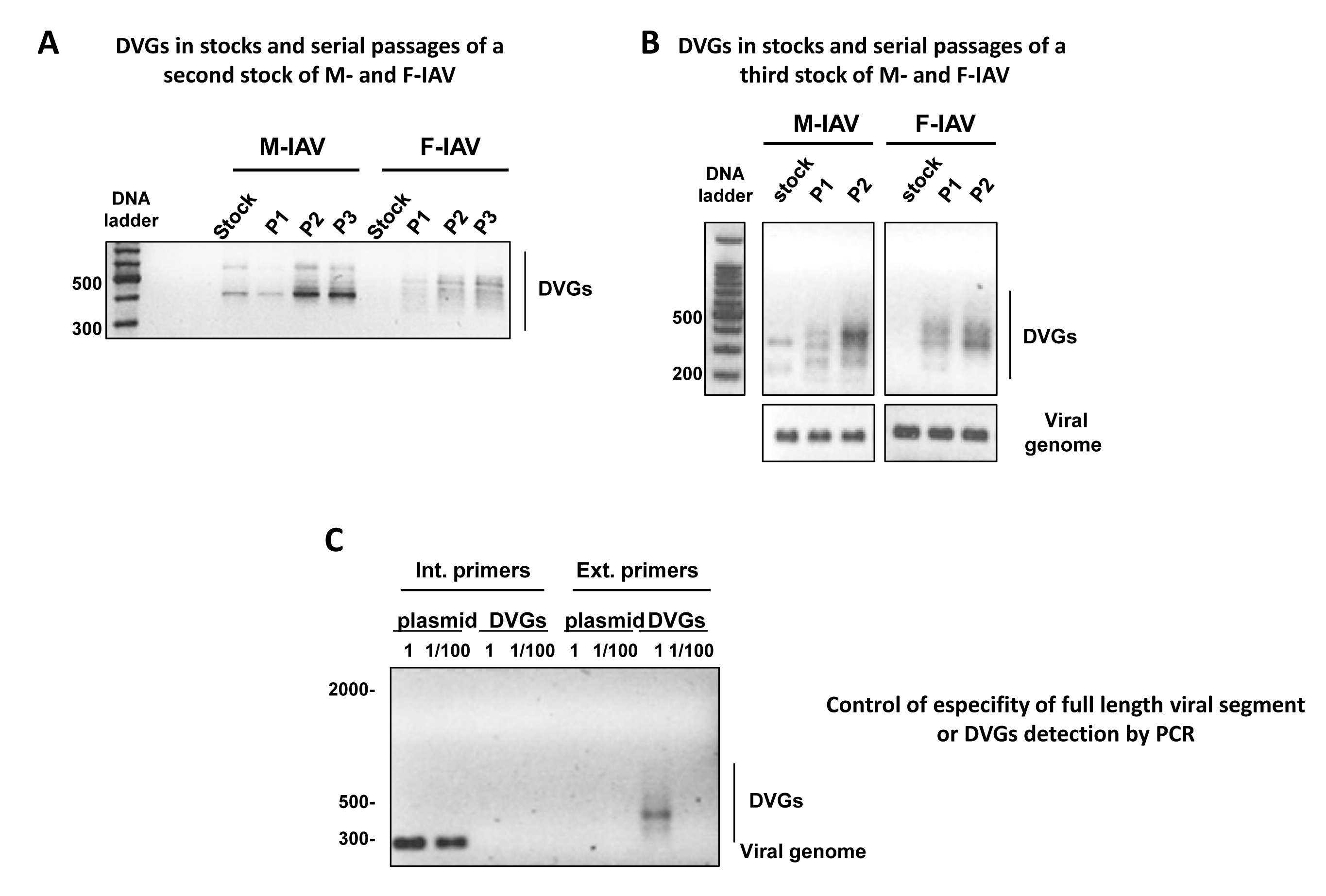

Supplement: S1 Fig — (A, B) Accumulation of DVGs after serial passages (P1, P2, P3) of two different M-IAV and F-IAV stocks. DNA ladder size indicated in nucleotides. (C) RT-PCR specificity controls. Primers and amplification conditions for internal fragment corresponding to full-length segment (Int primers) were used with a plasmid encoding the full-length PB2 segment (1 or 1/100 dilution) or with purified DVGs (1 or 1/100 dilution). The primers and RT-PCR conditions for DVGs amplification (Ext primers) were used with a plasmid encoding the full-length PB2 segment (1 or 1/100 dilution) or with purified DVGs (1 or 1/100 dilution). (TIF) [file ppat.1006650.s001.tif]

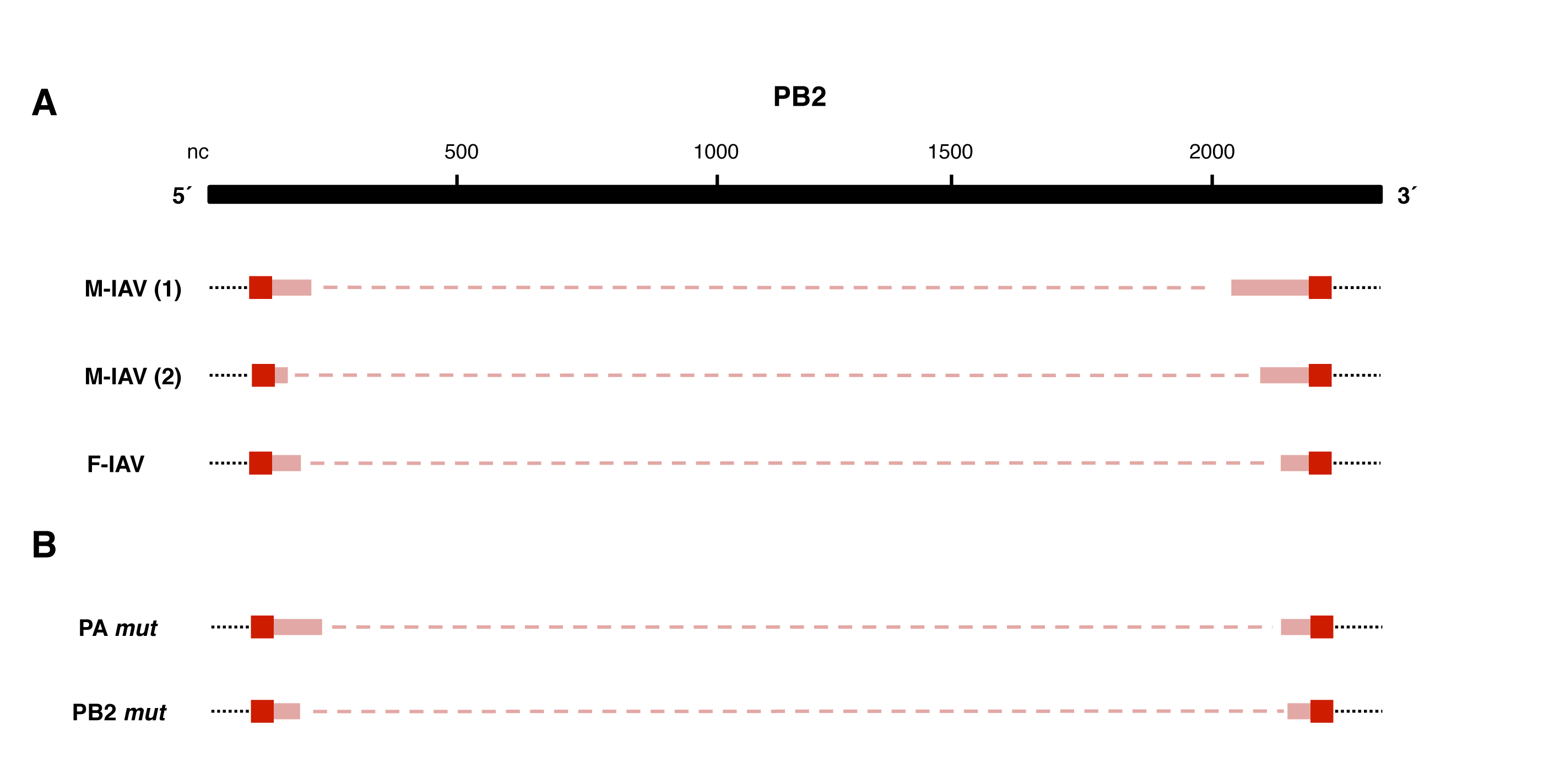

Supplement: S2 Fig — PCR amplified DVGs clones of PB2 segment from (A) M-IAV (clones 1 and 2) and F-IAV clinical isolates, or (B) PA mut and PB2 mut recombinant viruses are aligned with the A/Cal/04/09 reference sequence. Dotted lines denote UTR sequences, Red rectangles denote primers for DGs sequencing, Pink rectangles denote PB2 sequences. (TIF) [file ppat.1006650.s002.tif]

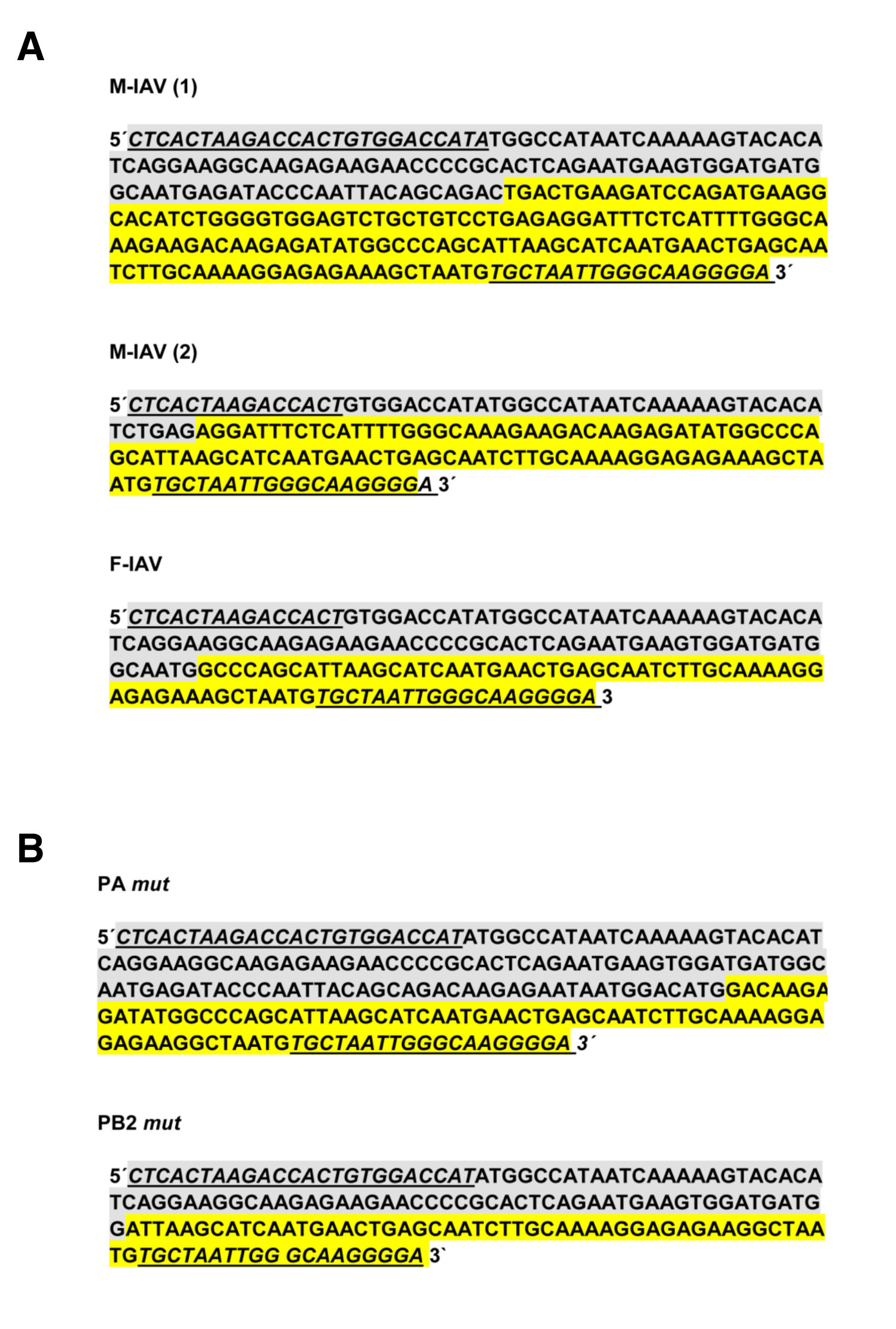

Supplement: S3 Fig — Sequence of the DVG clones corresponding to PB2 segment from (A) M-IAV (clones 1 and 2) and F-IAV clinical isolates or (B) PA mut and PB2 mut recombinant viruses. Underlined sequences represent the primers used for sequencing. Grey boxes come from the 5′end of the PB2 segment fused to the yellow boxes that come from the 3′end. (TIF) [file ppat.1006650.s003.tif]

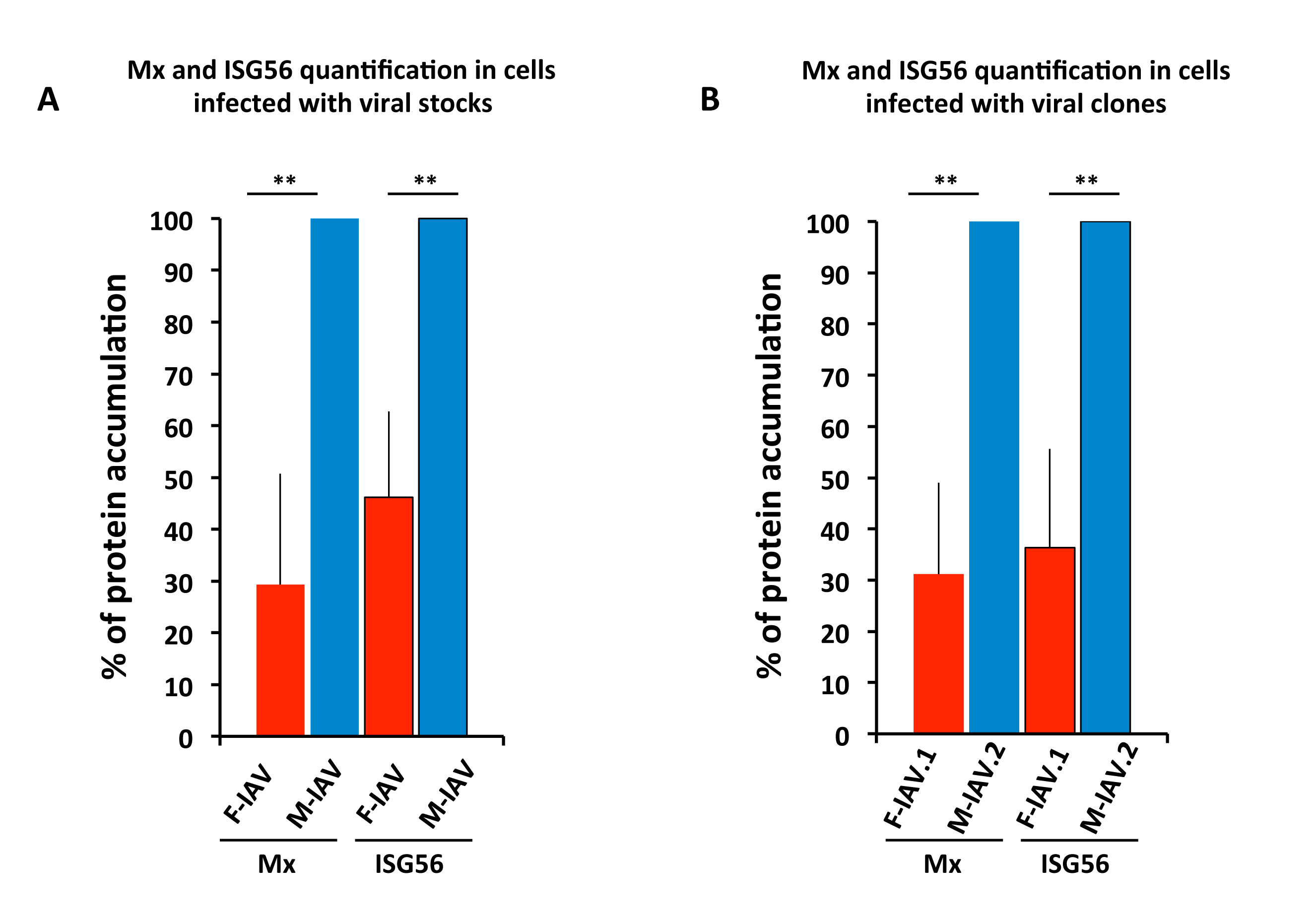

Supplement: S4 Fig — (A) Cultured human lung epithelial cells (A549) were infected with F- and M-IAV stocks or (B) with plaque purified clones of F- or M-IAV at moi 3. At 24 hours post-infection (hpi), samples were used to detect the indicated proteins by Western blot. MOCK cells treated with PBS as negative control and were used as background for quantitative analysis. β- actin antibody was used as loading control. Error bars indicate mean ± SD of three independent experiments (*p<0.05, **p<0.01 by t-Student test). (TIF) [file ppat.1006650.s004.tif]

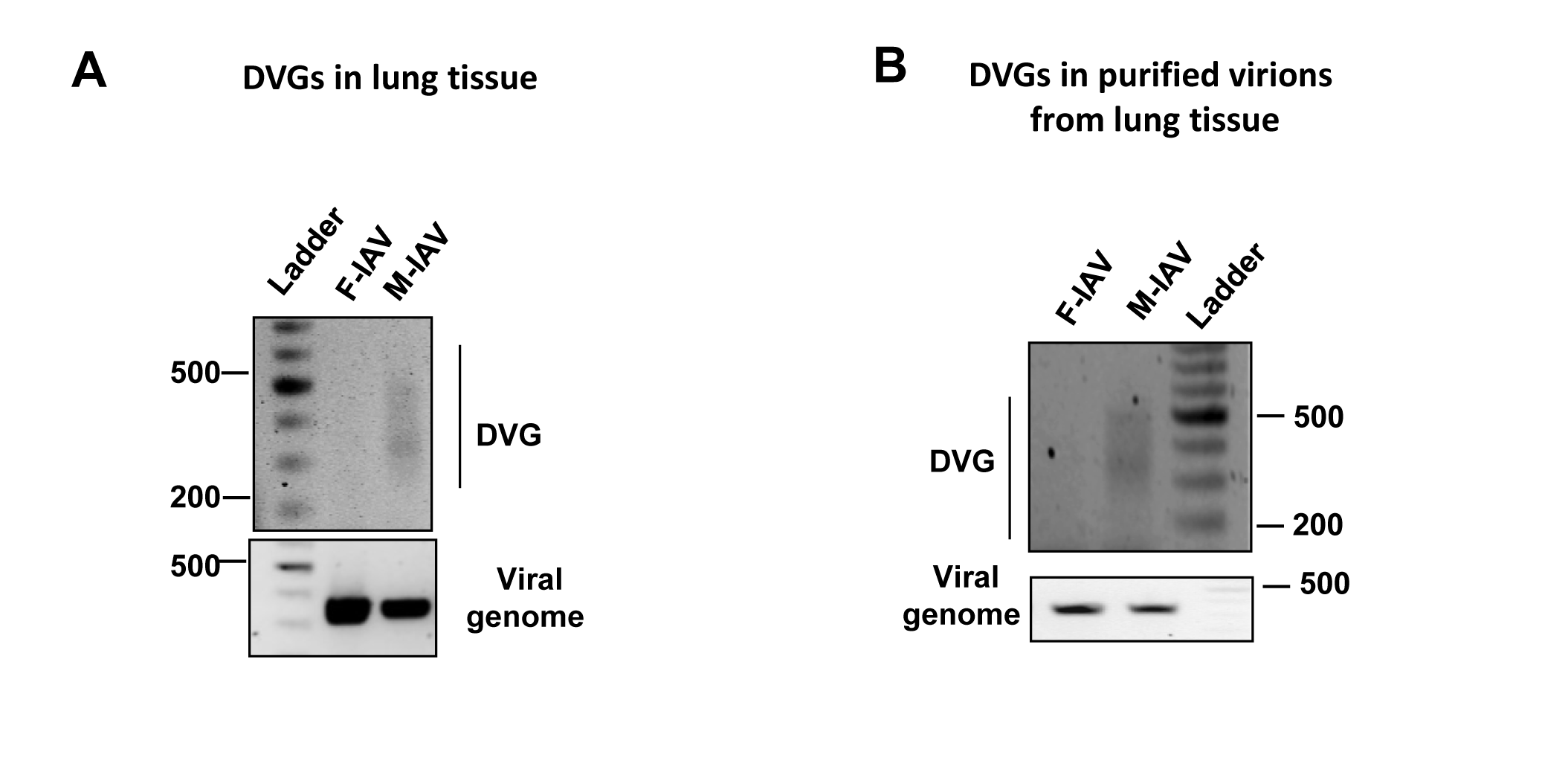

Supplement: S5 Fig — PCR detection of DVGs (top) or viral genome (bottom) of the PA segment in lung extracts (A) and in virions purified from lungs (B) of mice infected with F- or M-IAV isolates. DNA ladder size indicated in nucleotides. (TIF) [file ppat.1006650.s005.tif]

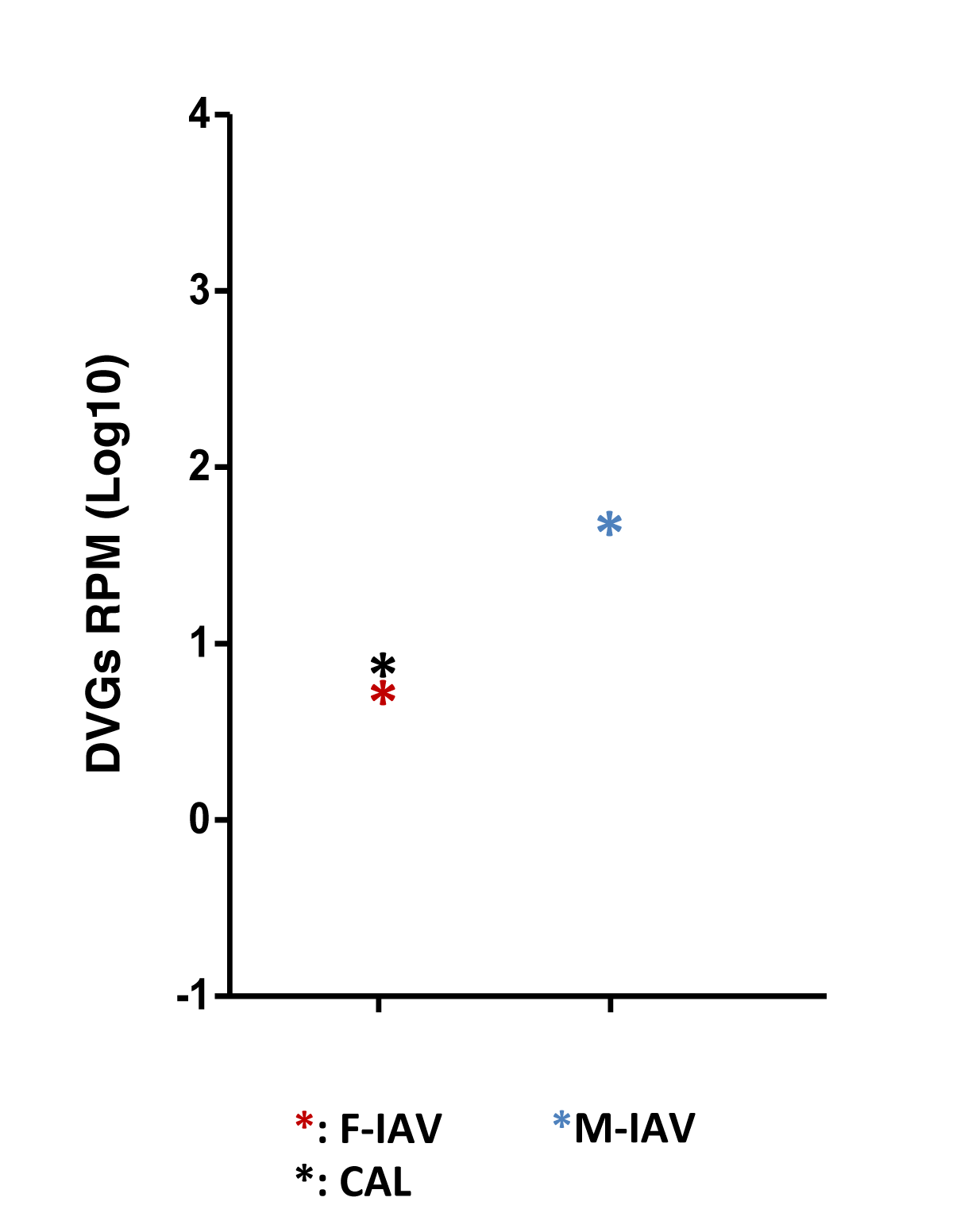

Supplement: S6 Fig — DVGs proportion in CAL recombinant virus and F- and M-IAV clinical isolates. CAL recombinant virus has been used as backbone for mutant viruses in the present study. Scatter plot representation of DVGs proportions, calculated as jumping reads/reads per million (RPM) that align the viral genome. Black indicates CAL wild-type recombinant virus, red indicates F-IAV,-Blue indicates M-IAV. (TIF) [file ppat.1006650.s006.tif]

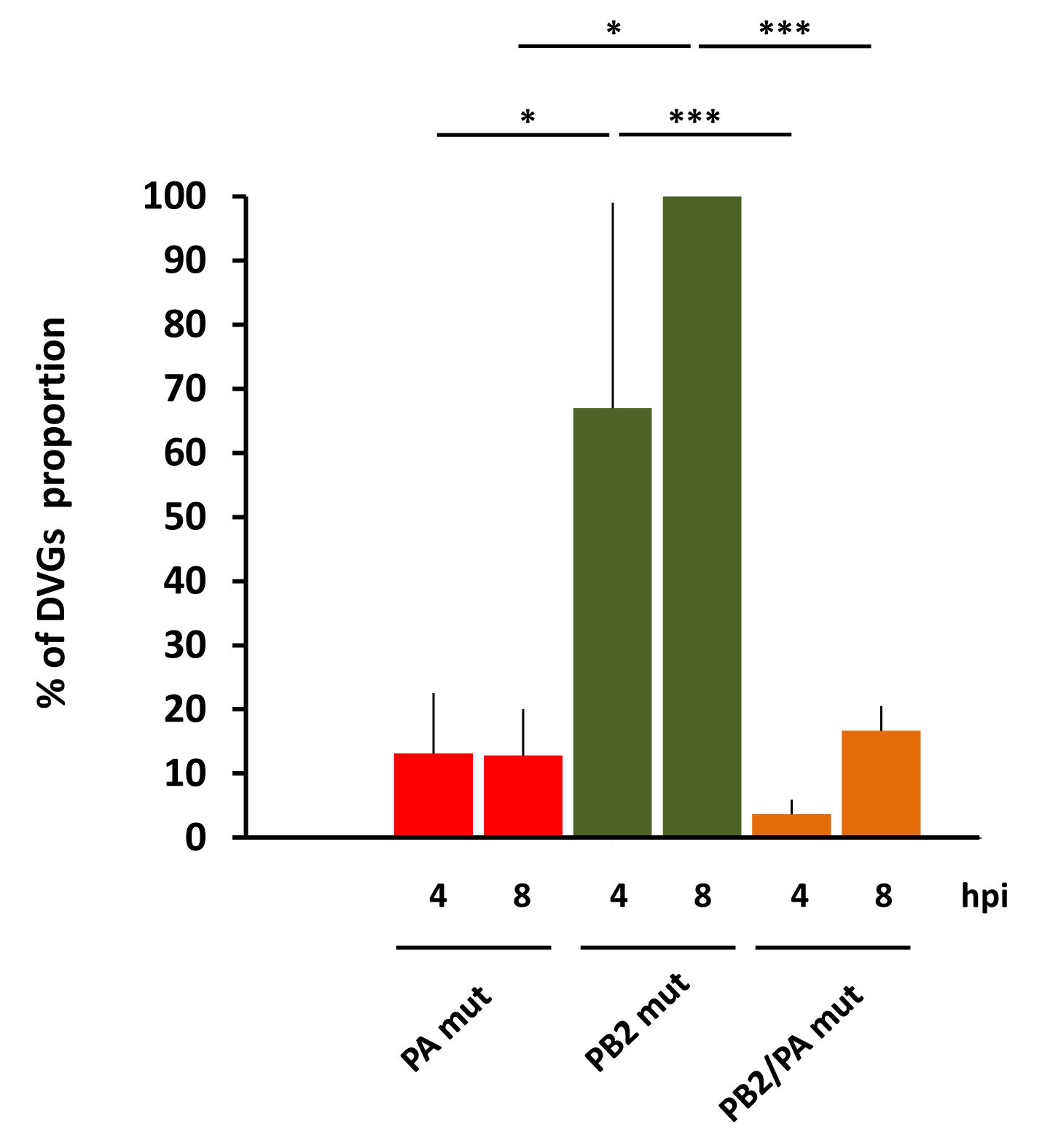

Supplement: S7 Fig — Cultured human lung epithelial cells (A549) were infected with indicated mutant viruses stocks at moi 1. Intracellular accumulation of DVGs was determined at indicated hours post-infection (hpi) as the ratio between DVGs and full length viral genomes (as shown in Fig 6). Error bars indicate mean ± SD of three independent experiments (*p<0.05, **p<0.01, ***p<0.001 by two-way ANOVA with Bonferroni post hoc test). (TIF) [file ppat.1006650.s007.tif]

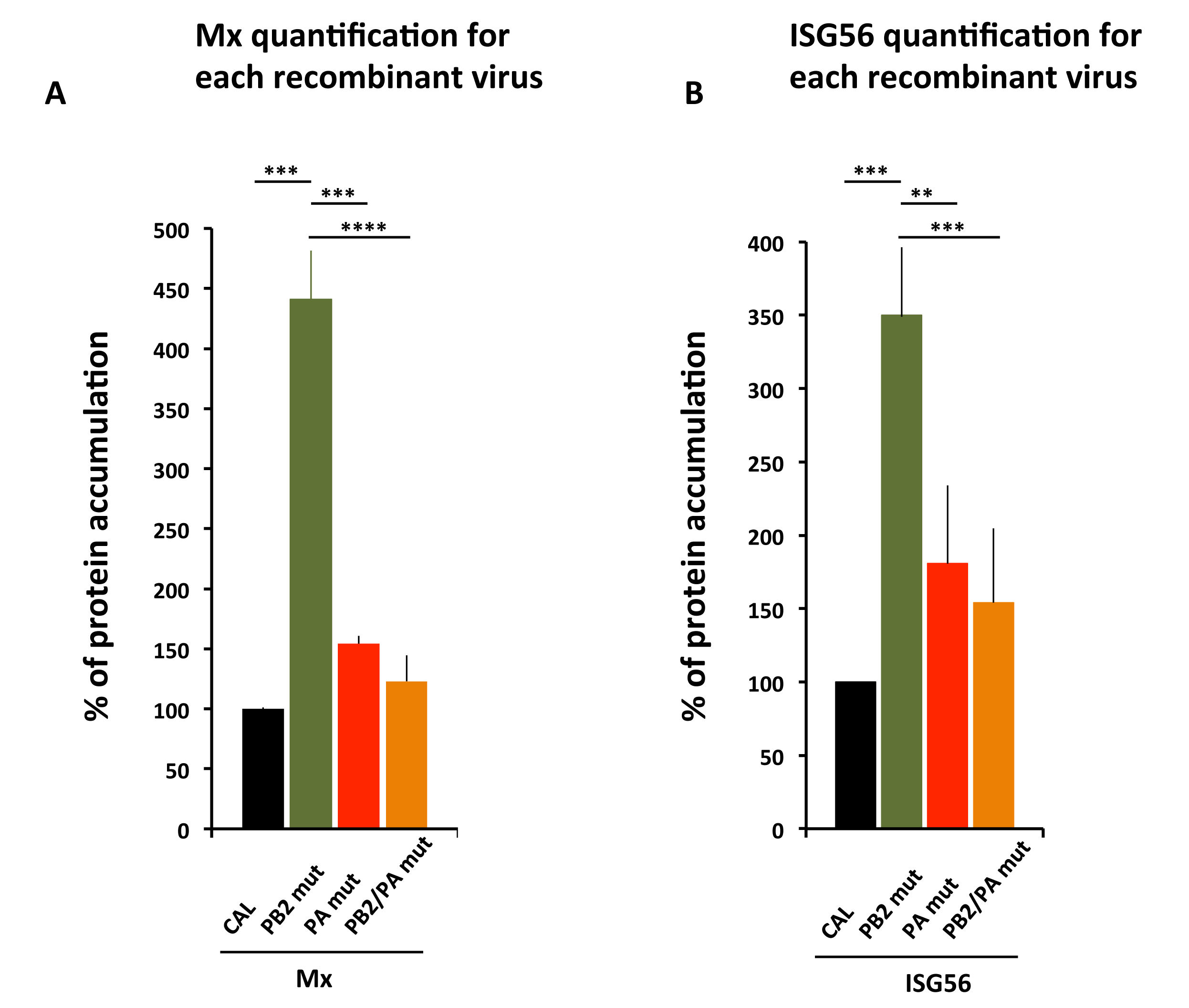

Supplement: S8 Fig — Cultured human lung epithelial cells (A549) were infected with CAL or mutant viruses stocks at moi 1. At 16 hours post-infection (hpi), samples were used to detect (A) Mx protein or (B) ISG56 protein by Western blot. MOCK cells treated with PBS as negative control and were used as background for quantitative analysis. β-actin antibody was used as loading control. Error bars indicate mean ± SD of three independent experiments (*p<0.05, **p<0.01, ***p<0.001 by two-way ANOVA with Bonferroni post hoc test). (TIF) [file ppat.1006650.s008.tif]

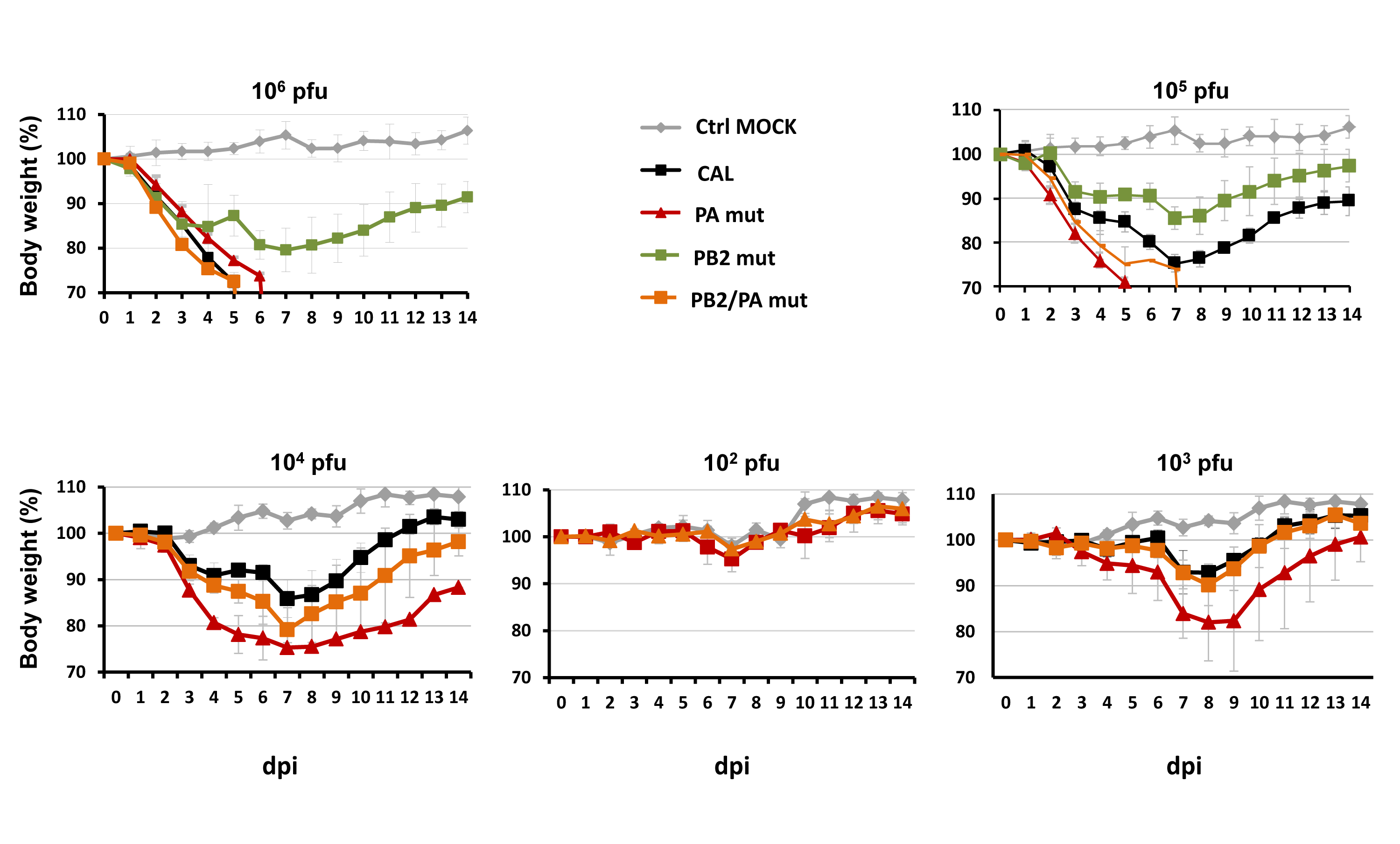

Supplement: S9 Fig — Mice (n = 5) were inoculated intranasally with different doses (106–102 pfu) of each CAL, PA mut, PB2 miut or PB2/PA mut recombinant virus or were mock-infected as control. Body weight for each group of animals was monitored daily for 14 days post-infection (dpi). (TIF) [file ppat.1006650.s009.tif]

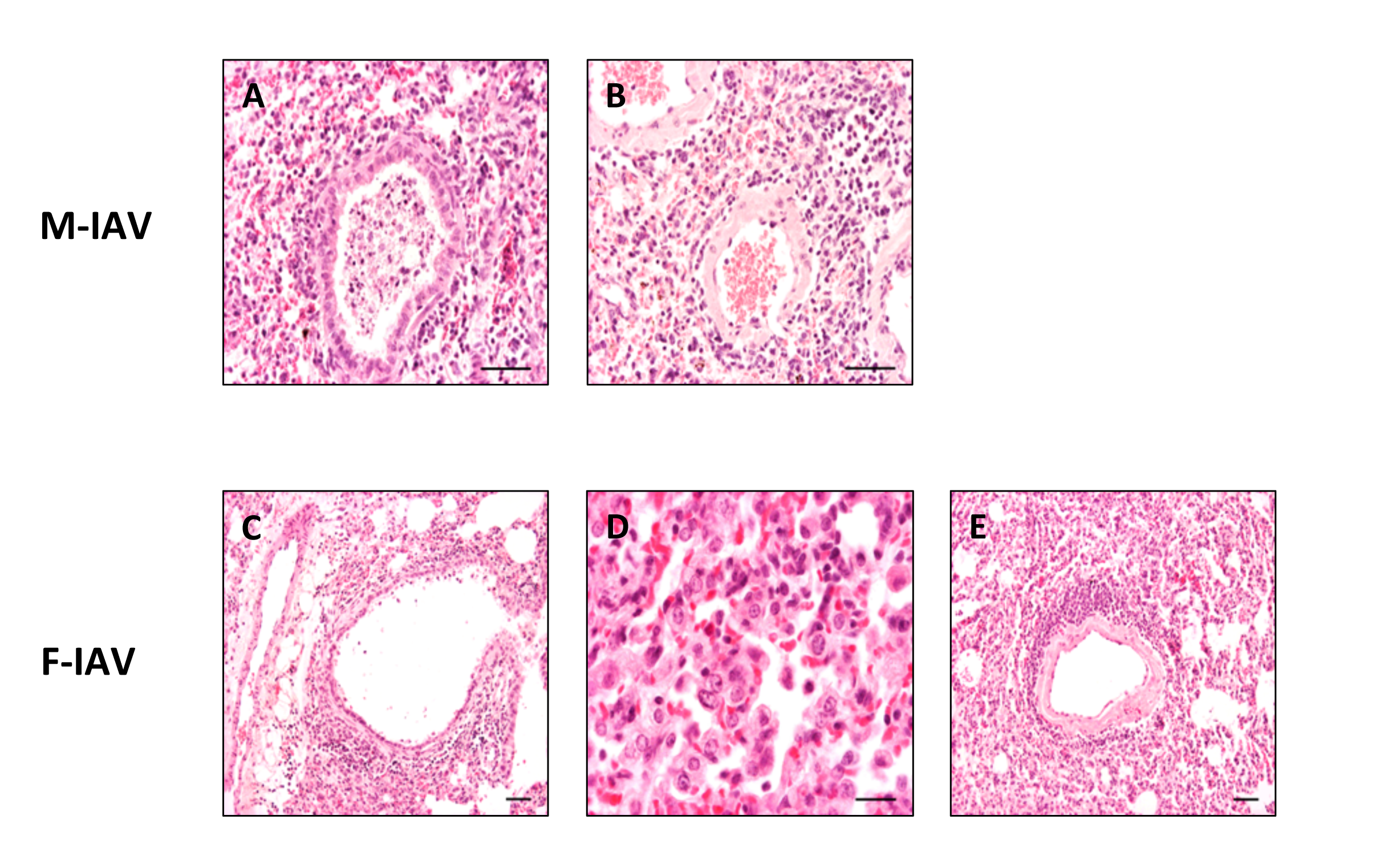

Supplement: S10 Fig — (A,B) Hematoxylin and eosin staining (H&E) of mice lung inoculated with M-IAV. (A) Congestion and diffuse lymphoid infiltrates in the interstitium. bar = 50 μm, (B) Mild perivasculitis: note mild inflammatory infiltrates of lymphocytes around a small arteriole and diffuse in the interstictial tissue. bar = 50 μm. (C-E); H&E of of mouse lung inoculated with F-IAV. (C) Moderate inflammatory infiltrates of lymphocytes around a dilated bronchiole. bar = 50 μm, (D) Severe hyperplasia of phagocytic cells in the interstitium. bar = 20 μm, (E) Mild perivasculitis: moderate amount of lymphoid cell infiltrates around a pulmonary arteriole. bar = 50 μm. The arrows show some examples of the lesion indicated in every case. (TIF) [file ppat.1006650.s010.tif]

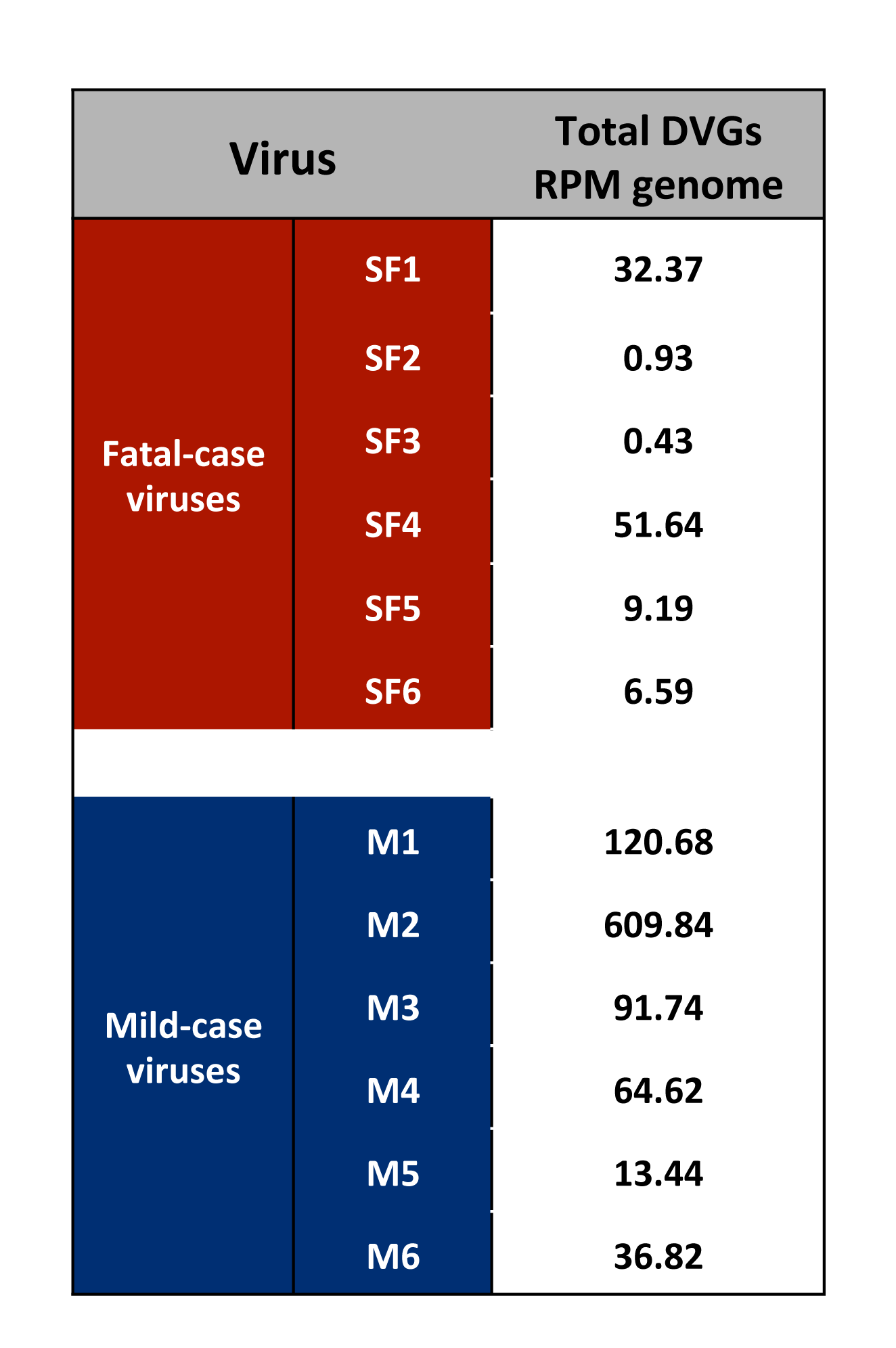

Supplement: S11 Fig — DVGs proportions, calculated as jumping reads per million (RPM) that align the viral genome, found in virions isolated from influenza A virus infected patients. Severe/fatal-case viruses, SF1-SF6; Mild-case viruses, M1-M6. (TIF) [file ppat.1006650.s011.tif]

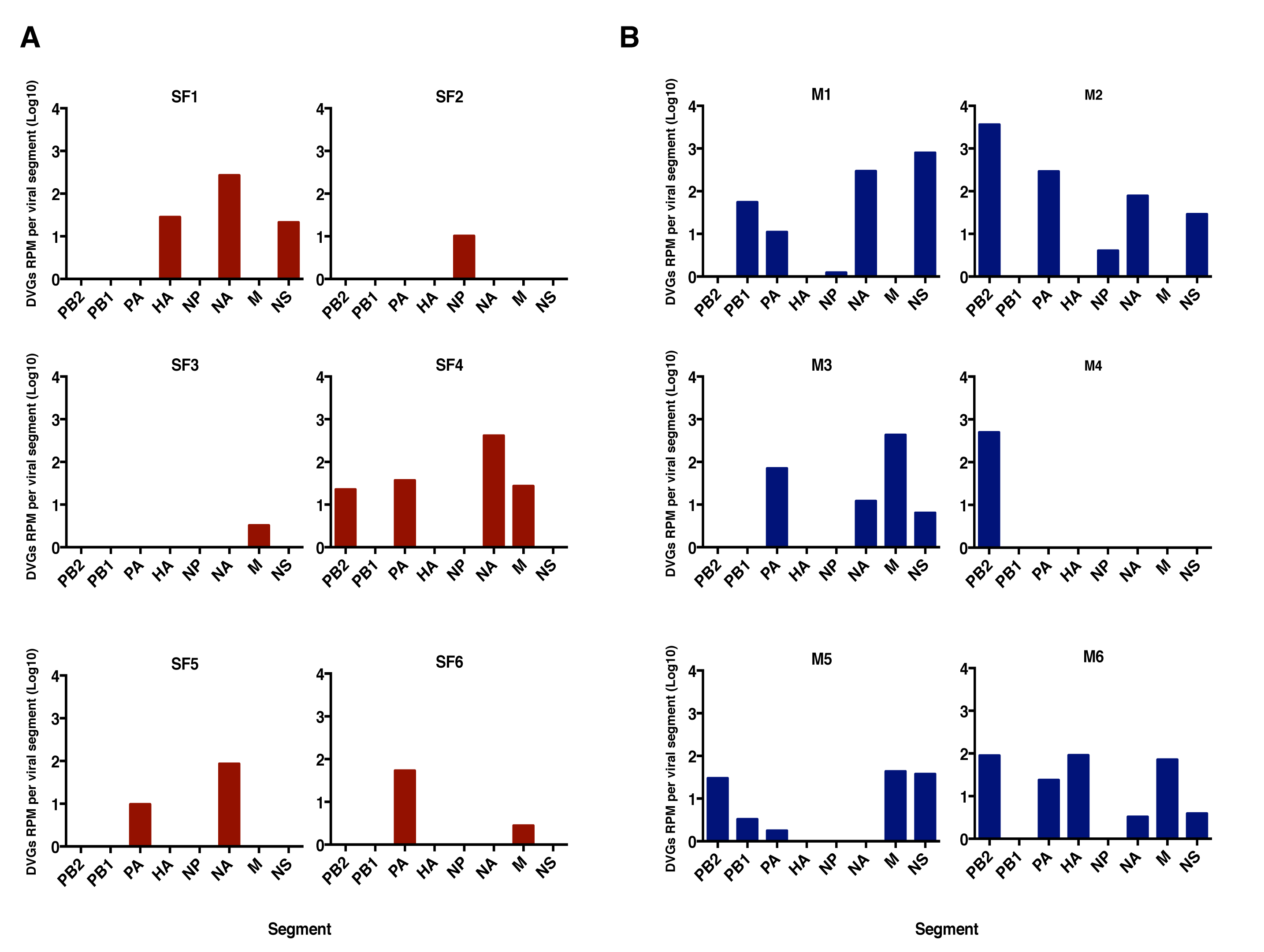

Supplement: S12 Fig — Log scale representation of DVGs distribution per segment calculated as jumping reads per million (RPM) that align each viral segment, analyzed in purified virions from A) Severe/fatal case viruses, SF1-SF6; B) mild-case viruses, M1-M6. Viral segments, PB1, PB2, PA, HA, NP, NA, M, NS. Note that these DVGs distributions are relative to all DVGs found in each virus, and their amounts are not strictly comparable from one virus to the others. (TIF) [file ppat.1006650.s012.tif]

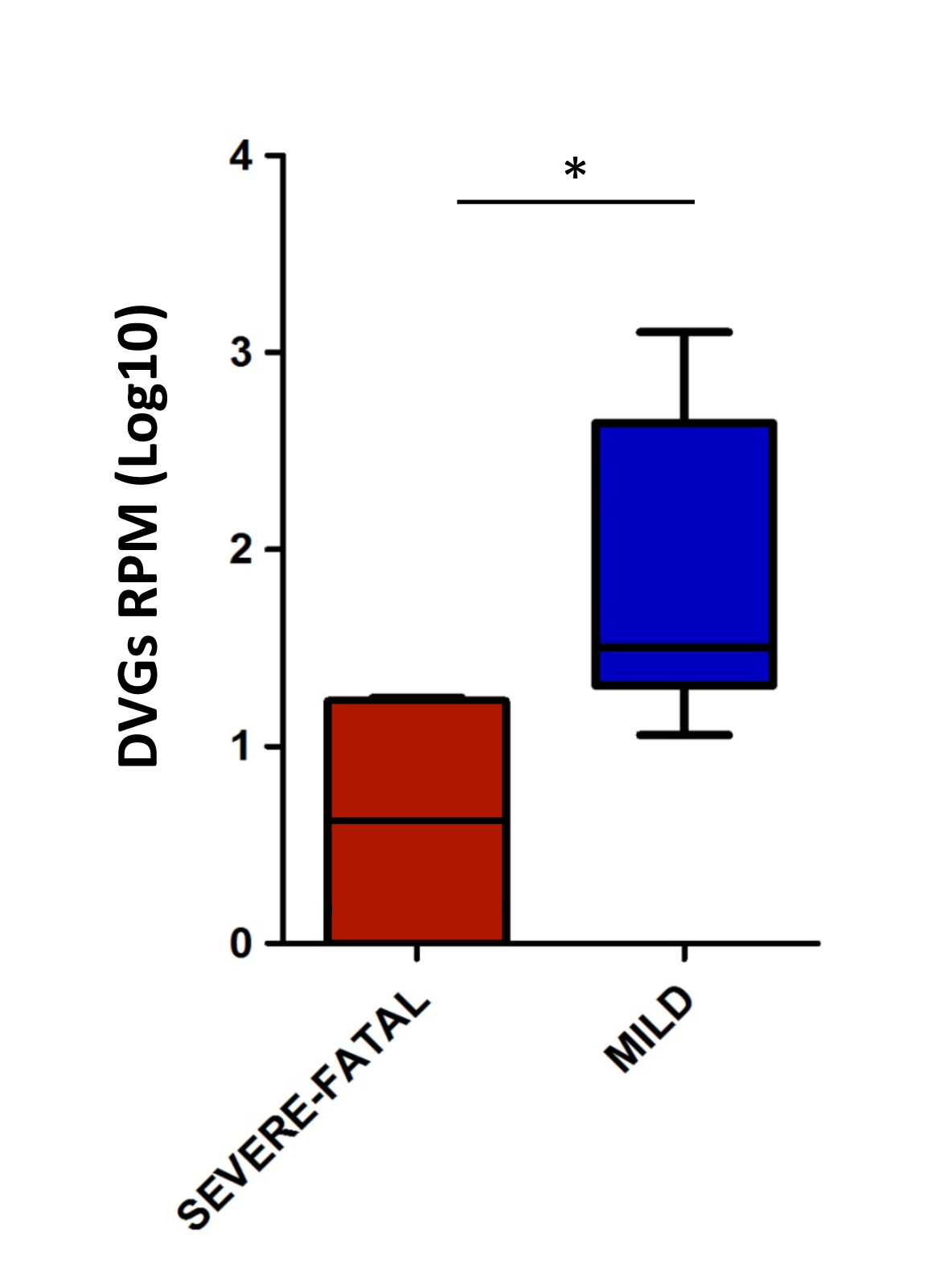

Supplement: S13 Fig — Scatter plot representation of DVGs proportions, calculated as jumping reads/reads per million (RPM) that align the viral genome, found in virions isolated from influenza A virus infected patients. Red and blue boxes indicate the interquartiles with values representing the intermediate 50% of the population. Severe/Fatal cases, n = 6; Mild cases, n = 6. Horizontal bars inside each box indicate the median value. Significance was determined by a two-tailed Mann-Whitney test; p = 0.0124 (* p <0.05). (TIF) [file ppat.1006650.s013.tif]

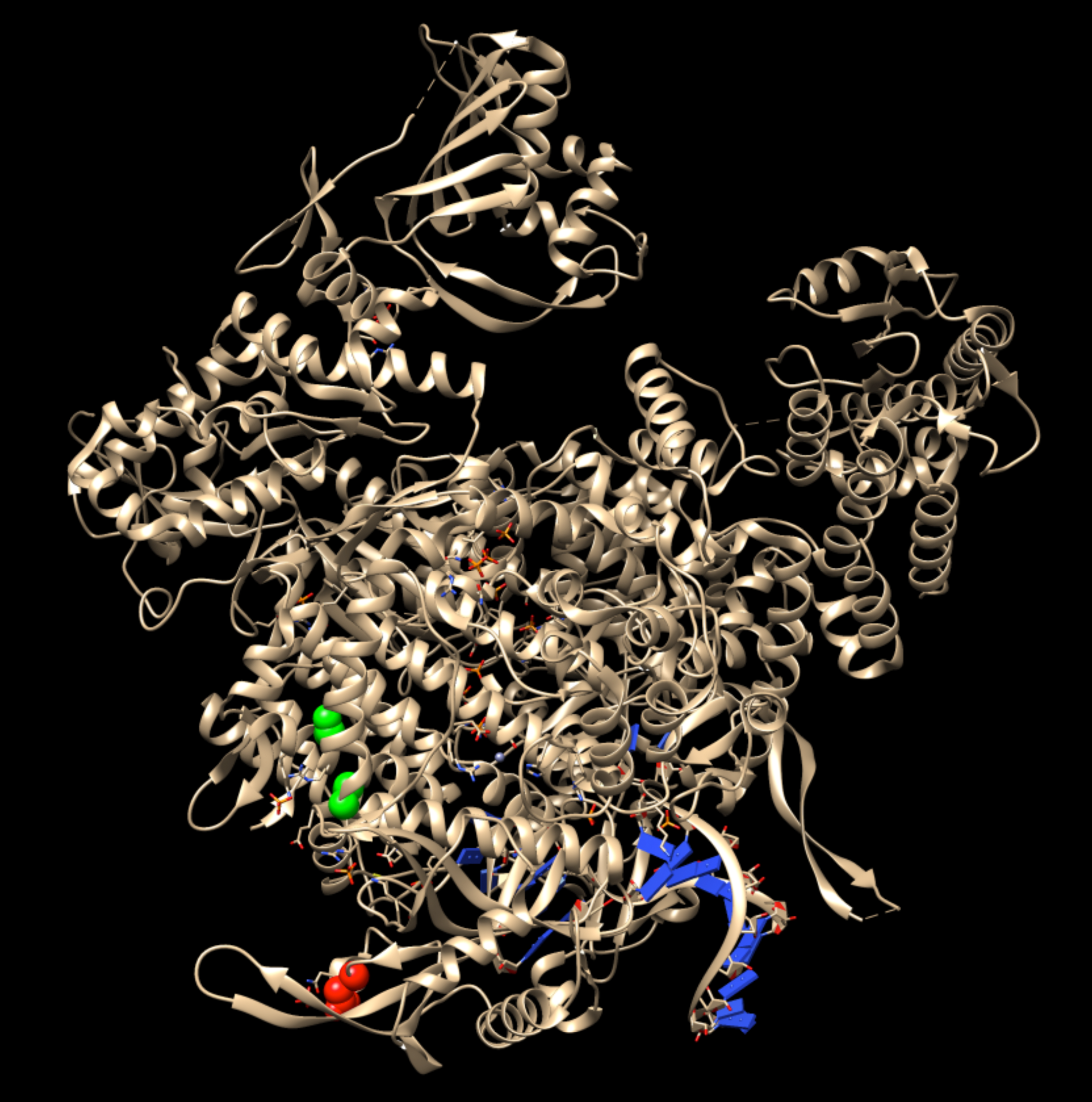

Supplement: S14 Fig — Structure of the viral polymerase complex in Protein Data Bank (PDB) under accession 4WSB has been used as template for structural localization of PA 529N (red) mutations with UCSF Chimera 1.10.2. PA 638A and 453R mutations previously described [Fodor et al;. 2003] are shown in yellow. RNA viral promoter is shown in blue. (TIF) [file ppat.1006650.s014.tif]

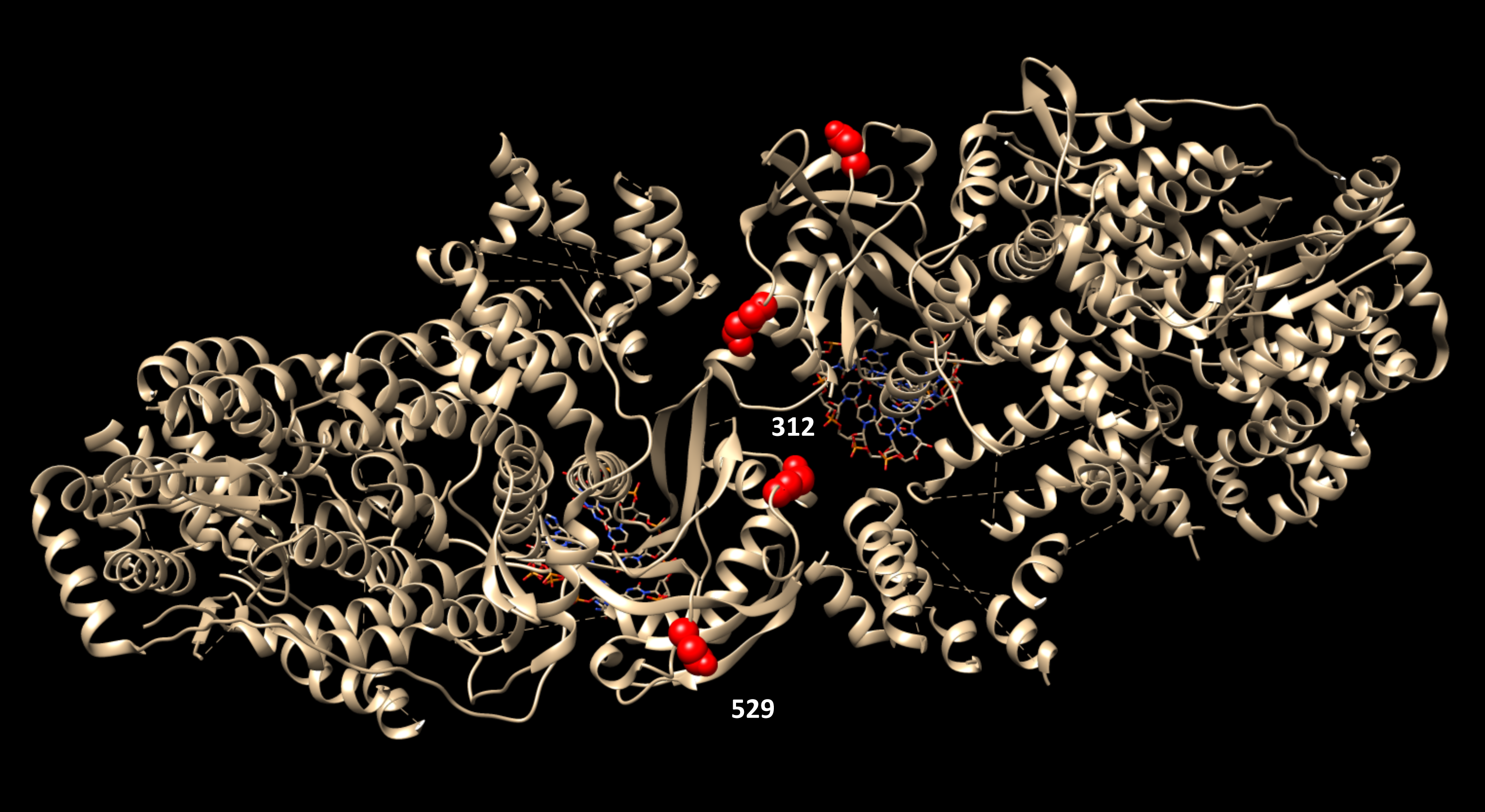

Supplement: S15 Fig — Structure of the dimer polymerase complex in Protein Data Bank (PDB) under accession 3J9B has been used as template for structural localization of PA mutations with UCSF Chimera 1.10.2. PA mutations described in the severe-fatal case viruses in the present study (PA 529 and PA 312) are shown in red. RNA viral promoter is shown in blue. (TIF) [file ppat.1006650.s015.tif]

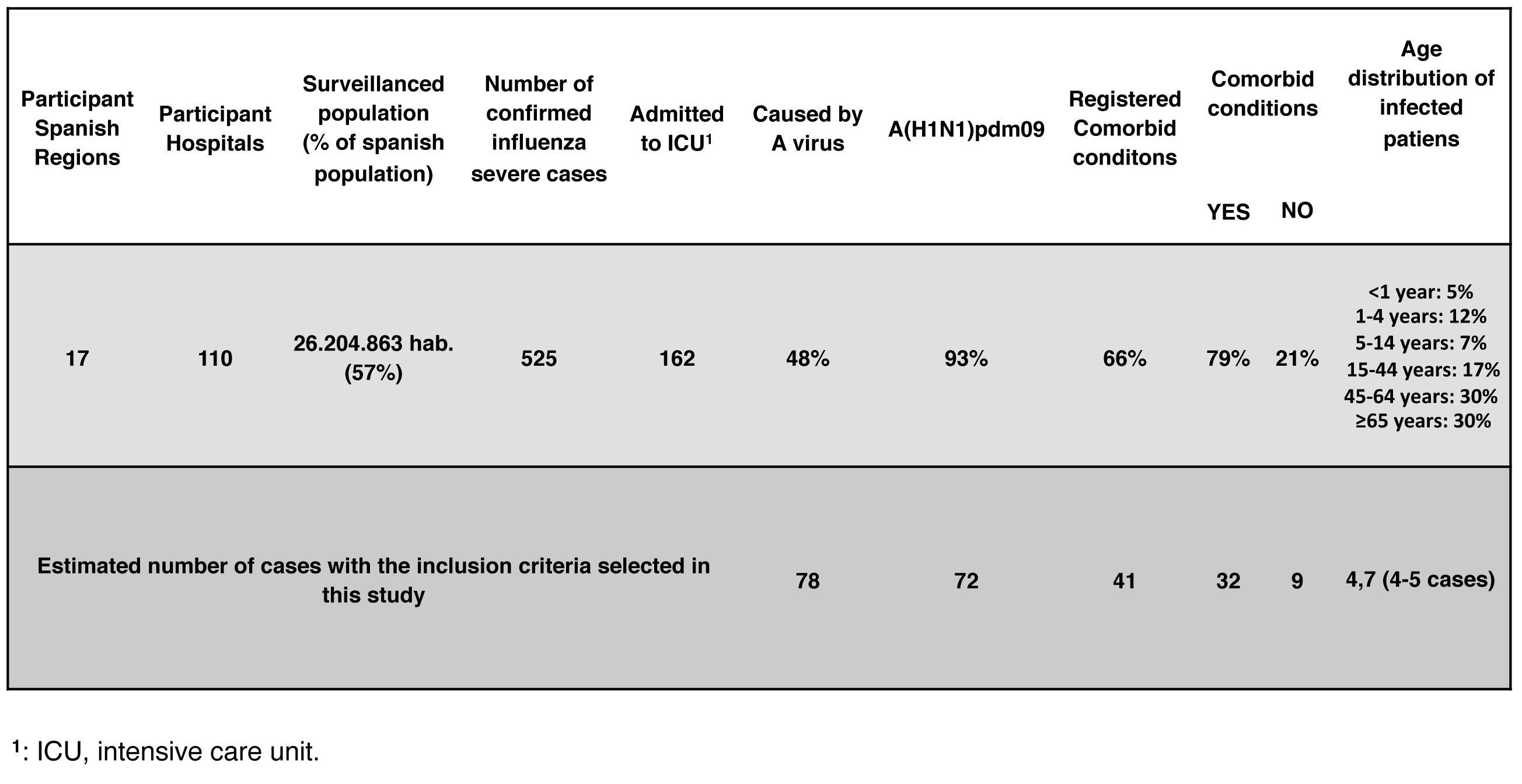

Supplement: S2 Table — Epidemiological data, including comorbidities information, reported by the National Epidemiological Influenza Center are shown for the 2012–2013 Spanish influenza seasons. (TIF) [file ppat.1006650.s017.tif]

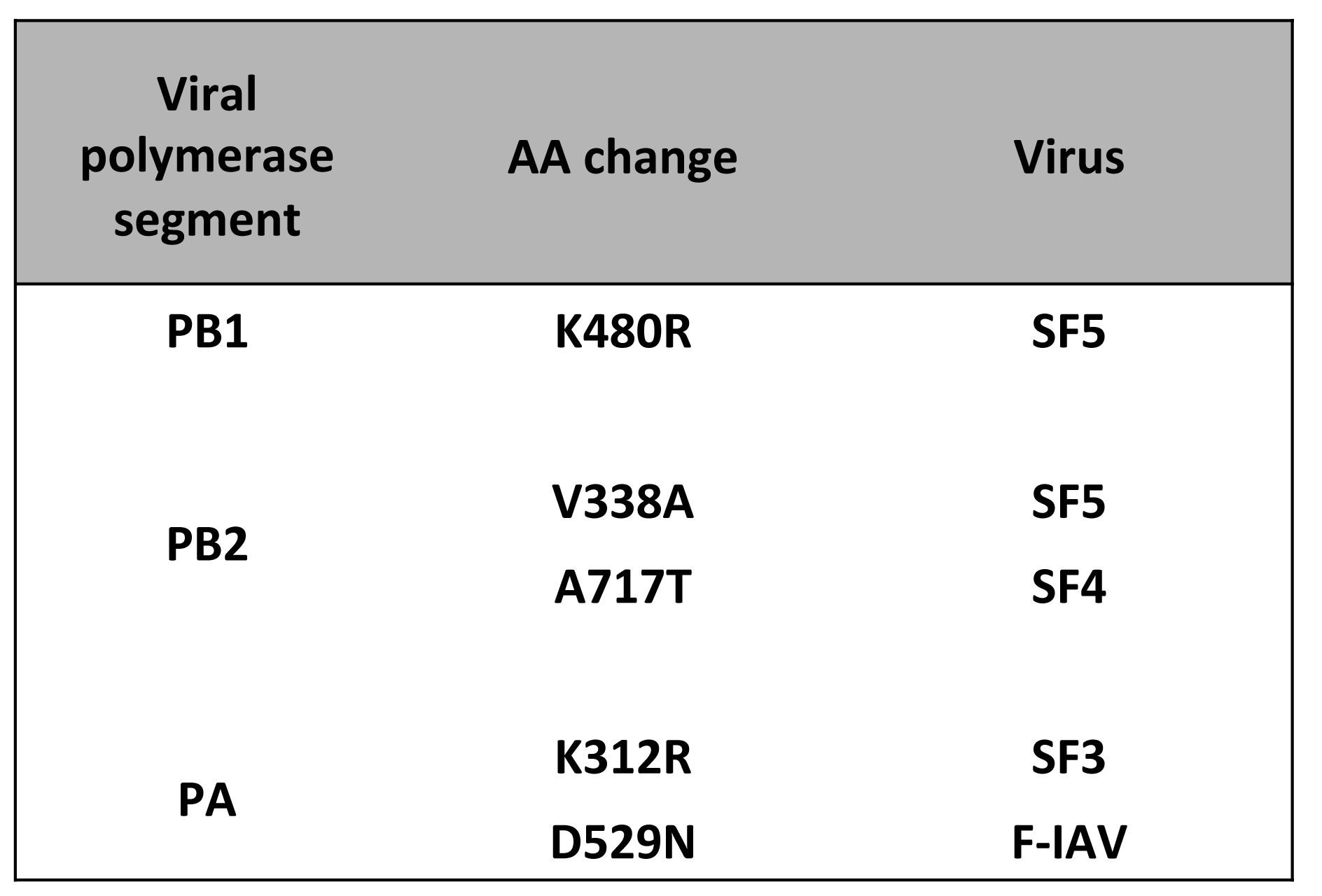

Supplement: S3 Table — (TIF) [file ppat.1006650.s018.tif]
